# Macrophage migration inhibitory factor (MIF) suppresses mitophagy through disturbing the protein interaction of PINK1-Parkin in sepsis-associated acute kidney injury

**DOI:** 10.1038/s41419-024-06826-z

**Published:** 2024-07-02

**Authors:** Tianlong Li, Jiachen Qu, Chang Hu, Jingjing Pang, Yaoyao Qian, Yiming Li, Zhiyong Peng

**Affiliations:** Department of Critical Care Medicine, Zhongnan Hospital of Wuhan University, Wuhan, Hubei province 430071 China

**Keywords:** Acute kidney injury, Mitophagy

## Abstract

Damage to renal tubular epithelial cells (RTECs) signaled the onset and progression of sepsis-associated acute kidney injury (SA-AKI). Recent research on mitochondria has revealed that mitophagy plays a crucial physiological role in alleviating injury to RTECs and it is suppressed progressively by the inflammation response in SA-AKI. However, the mechanism by which inflammation influences mitophagy remains poorly understood. We examined how macrophage migration inhibitory factor (MIF), a pro-inflammatory protein, influences the PINK1-Parkin pathway of mitophagy by studying protein–protein interactions when MIF was inhibited or overexpressed. Surprisingly, elevated levels of MIF were found to directly bind to PINK1, disrupting its interaction with Parkin. This interference hindered the recruitment of Parkin to mitochondria and impeded the initiation of mitophagy. Furthermore, this outcome led to significant apoptosis of RTECs, which could, however, be reversed by an MIF inhibitor ISO-1 and/or a new mitophagy activator T0467. These findings highlight the detrimental impact of MIF on renal damage through its disruption of the interaction between PINK1 and Parkin, and the therapeutic potential of ISO-1 and T0467 in mitigating SA-AKI. This study offers a fresh perspective on treating SA-AKI by targeting MIF and mitophagy.

## Introduction

Sepsis triggers a systemic inflammatory response syndrome and typically leads to organ damage, often affecting the kidneys, lungs, and liver [1]. Patients diagnosed with sepsis-associated acute kidney injury (SA-AKI) experience symptoms including swelling, reduced urine output, elevated creatinine levels, acid-base imbalances, and electrolyte disruptions. Over time, many of these patients progress to chronic kidney injury (CKD) or even kidney failure [2, 3]. The symptoms mentioned above could initiate due to injury in renal tubular epithelial cells (RTECs) induced by lipopolysaccharide (LPS). While the primary causes of SA-AKI and RTECs injury remain unidentified, they are closely linked to mitochondrial dysfunction [4, 5].

Mitophagy, a crucial biological process of mitochondria, relies on the functioning of autophagosomes and lysosomes. It plays a significant role in degrading damaged mitochondria and maintaining the balance of mitochondrial biogenesis [6]. Within a low-stress intracellular environment, mitophagy is primarily governed by the PTEN-induced putative kinase 1 (PINK1) - Parkin RBR E3 ubiquitin-protein ligase (Parkin) pathway [7]. This pathway serves as a vital therapeutic target for mitochondrial diseases. In conditions such as Alzheimer’s disease, overexpression of PINK1 has been shown to mitigate oxidative damage and abnormal mitochondrial dynamics by expediting Parkin recruitment to impaired mitochondria [8]. However, in cases of SA-AKI, persistent inflammation exacerbates the accumulation of reactive oxygen species (ROS) and suppresses mitophagy [9]. While it has been recognized that deficiency in the PINK1 or PRKN (the gene name of Parkin) genes can lead to severe kidney injury and cellular apoptosis, the cause of mitophagy suppression by the inflammatory response remains elusive [10]. Parkin is susceptible to degradation via the ubiquitination pathway, resulting in a decline in expression after recruitment by PINK1 [11, 12]. The use of a mitochondrial uncoupler (carbonyl cyanide 3-chlorophenylhydrazone, CCCP) rapidly induces acute depolarization of the mitochondrial membrane potential (ΔΨm) and initiates mitophagy, which also hastens Parkin consumption. Typically, the restoration of ΔΨm occurs through mitophagy following the cessation of short-term CCCP induction [13]. If there is a continuous deterioration in the dissipation of ΔΨm, it indicates that the PINK1-Parkin pathway of mitophagy has been completely exhausted. Hence, it becomes imperative to explore alternative approaches in SA-AKI models.

Macrophage migration inhibitory factor (MIF) is a versatile protein weighing about 12.5 KDa. It is consistently expressed by epithelial cells, endothelial cells, and macrophages to regulate various cellular processes such as proliferation, programmed cell death, signal transduction, and RNA transcription [14]. MIF is primarily known for its pro-inflammatory role and has garnered significant attention in this regard. Recent research has revealed that serum MIF levels can forecast the onset of SA-AKI in its early phases, with elevated levels indicating greater severity of SA-AKI and poorer patient outcomes [15, 16]. Under LPS stimulation, the expression of MIF in RTECs increased, exacerbating the formation of NLRP3 inflammasomes and leading to RTECs injury in SA-AKI [17]. Inhibiting MIF effectively suppressed the activation of NLRP3 inflammasomes [18, 19], the buildup of ROS, and the activation of the NFκB pathway [20–22]. These effects are akin to those achieved by the PINK-Parkin pathway of mitophagy [23–25]. Furthermore, preliminary verification via quantitative proteomics indicated that MIF within the nucleus pulposus cell regulated the ATP biosynthetic process and oxidative phosphorylation, processes closely linked to mitochondrial function [26]. MIF could be regarded both as a biomarker and a potential therapeutic target for SA-AKI. Nevertheless, the mechanism by which MIF influences mitophagy in SA-AKI remains poorly understood.

In our study, SA-AKI models were established both in vivo and in vitro. In vivo models were created using mice that underwent cecum ligation puncture (CLP) surgery, while in vitro models involved human kidney-2 (HK-2) cells stimulated with LPS. Mitochondrial membrane potential (ΔΨm), mitophgosomes and the cell counting of mitophagosomes formation were assessed by JC-1 staining, transmission electron microscope (TEM), and immunofluorescence respectively. Recombinant human MIF (rhMIF) was used to determine the functional localization. Mitophagy inducers, including CCCP, olaparib (OLA), and T0467, were utilized to assess the inhibitory effect of inflammation on mitophagy and their potential therapeutic feasibility in SA-AKI. Additionally, changes in the expression of core proteins were evaluated under MIF inhibition or overexpression. Furthermore, the evidence of the protein-protein interactions (PPIs) was found in the BioGRID database. The mitophagy-related proteins capable of interacting with MIF were identified by immunoblots, co-immunoprecipitation, and confocal imaging. This relationship between MIF and the core proteins was confirmed by the transient transfection of siRNA. Subsequently, the efficacy of potential therapeutic agents was validated in vivo by assessing mitophagy-related indices, renal function (creatinine and blood urea nitrogen levels), and the 7-day survival status of SA-AKI mice.

## Materials and methods

### Establishment of SA-AKI mice model

In this research, we utilized the CLP surgery method to establish a mouse model of SA-AKI. Ten-week-old male C57BL/6 mice weighing between twenty to thirty grams underwent CLP surgery after an 8-h period of food and water deprivation. Prior to the operation, the mice received pre-treatment via intraperitoneal injection of drugs (ISO-1, T0467), while the CLP group received solvent (10% dimethylsulfoxide, DMSO). A midline incision shorter than one cm was made in the abdomen to expose the distal end of the cecum. The cecum was ligated with 4-0 silk at the midpoint, and a single gentle puncture was made between the ligature and the tip of the cecum in a mesenteric-to-antimesenteric direction. Subsequently, the cecum was returned to the abdominal cavity and the incision was sutured. Mice undergoing CLP surgery were revived by subcutaneously injecting pre-warmed 0.9% saline (50 μl/g body weight). The sham group mice underwent laparotomy and received fluid resuscitation but did not undergo CLP surgery. Blood samples and both kidneys were collected 24 h post surgery. For the 7-day observation period, mice were administered buprenorphine twice daily for postoperative analgesia. The mouse models in our study strictly adhered to a standardized CLP procedure [27].

### Animal experimental ethics

The C57BL/6 mice were procured from the GemPharmatech laboratory in Shanghai, China. All mice were housed and operated on in a sterile environment. They were provided with unrestricted access to food and water and were housed in standard cages with a 12-h light-dark cycle. All animal experiments conducted in this study adhered to the protocols approved by the Animal Ethics Committee of the Center for Animal Experiment at Wuhan University.

### Reagents and cell lines

HK-2 cells were purchased from the Cell Bank of the Chinese Academy of Sciences (Shanghai, China, RRID: CVCL_0302) and cultured in a mixture of 90% DMEM/F12 (ThermoFisher Scientific, 11330032) and 10% fetal bovine serum (FBS, Procell, 164210) in a cell incubator. Stringent protocols were followed for operating procedures and managing reagents, with regular monitoring and detection of cell contamination conducted monthly.

Murine primary renal tubular epithelial cells (mPTECs) were isolated from the kidneys of C57BL/6 mice through a process involving the removal and mincing of the kidney tissue, followed by digestion with a collagenase and dispase suspension. Subsequently, the tissue fragments were filtered through mesh sizes of 100, 70, and 40 μm to isolate the mPTECs. The isolated mPTECs were then cultured in DMEM/F12 supplemented with 10% FBS.

Various molecules, including recombinant human protein MIF (rhMIF, 10 μg/ml, MedChemExpress, HY-P7387), CCCP (10 μM, MedChemExpress, HY-100941), OLA (2 μM, MedChemExpress, HY-10162), ISO-1 (20 μM, MedChemExpress, HY-16692), and T0467 (5 μM, MedChemExpress, HY-139308), were individually co-incubated with HK-2 cells [17, 28–31]. Additionally, HK-2 cells were exposed to LPS challenge (10 μg/ml, InvivoGen, tlrl-eblps) [32]. Cell samples were collected at different time points (0, 12, 24, and 48 h) post-LPS stimulation. Furthermore, the following primary antibody reagents were employed in the immunological experiments: MIF antibody (RRID: AB_2788655), Caspase3 (Casp3) antibody (RRID: AB_10733244), cleaved-caspase3 (cleaved-casp3) antibody (RRID: AB_3073913), PINK1 antibody (RRID: AB_2879244), Parkin antibody (RRID: AB_2882028), voltage-dependent anion channel (VDAC1) antibody (RRID: AB_2881725), AMPK antibody (RRID: AB_2835253), phosphorylated AMPK (P-AMPK) antibody (RRID: AB_2834865), glyceraldehyde-3-phosphate dehydrogenase (GAPDH) antibody (RRID: AB_2107436), and β-Actin antibody (RRID: AB_2923704).

### Transfection of HK-2 cells

The transient transfection of HK-2 cells with siRNA (50 nM) was carried out using Lipofectamine™ 3000 Transfection Reagent (ThermoFisher Scientific, L3000150) for 6 h. Stable transfection of HK-2 cells was achieved using lentivirus provided by Genepharma Corporation (Shanghai, China). The lentivirus contained gene sequences for puromycin resistance, which were inserted into the lentiviral gene sequences. HK-2 cells were co-incubated with the lentivirus for 2 days. Once the cell density reached 80%, puromycin was added to the culture medium to screen out HK-2 cells with successful transfection. The sense sequences for si-*PINK1* were 5′-GCCAUCUUGAACACAAUGA-3′, for si-*PRKN* were 5′-GGA UCAGCAGAGCAUUGUU-3′, and for KD-*MIF* lentivirus were 5′-TGGACA GGGTCTACATCAACTTCAAGAGAGTTGATGTAGACCCTGTCCTTTTTTC-3′. The OE-MIF lentivirus utilized the EF1a promoter to drive the transcription of the *MIF* gene sequence and achieve MIF overexpression.

### Histology, immunohistochemistry and renal function

The kidney samples from mice were fixed in 4% paraformaldehyde and embedded in 10% paraffin. The paraffin-embedded kidney tissue blocks were sectioned into 3 μm slices for hematoxylin and eosin (H&E) staining and immunohistochemical staining. For immunohistochemical staining, the sections were incubated with MIF antibody or cleaved-casp3 antibody overnight at four degrees Celsius. Images of both H&E and immunohistochemical staining were captured and analyzed using CaseViewer 2.4 software.

Blood samples were collected in heparin tubes and allowed to stand motionless at four degrees Celsius for ten minutes. They were then centrifuged at the same temperature and layered. The plasma in the supernatant was collected and stored at minus eighty degrees Celsius. Creatinine (Cre, Jiancheng Bioengineering Institute, C011-2-1) and blood urea nitrogen (BUN, Jiancheng Bioengineering Institute, C013-2-1) levels were measured using commercial reagent kits, and the data were recorded using a microplate reader (PerkinElmer, USA).

### Mitochondrial isolation

A batch of cells or a segment of renal tissues was incubated with a mitochondria isolation reagent (MedChemExpress, HY-K1060, and HY-K1061) on ice and subsequently homogenized. Gradient centrifugation was employed to separate organelles at 600 × *g*, enrich mitochondria at 4000 × *g*, and purify cytoplasm at 12,000 × *g*. Mitochondria were identified through immunoblotting and TEM analysis.

### Immunofluorescence

Renal tissue sections or cell slides underwent dewaxing and hydration using xylene and ethanol. Subsequently, they were immersed in antigen retrieval buffer, boiled for twenty minutes, and left to cool naturally. To block peroxidase activity, a 3% hydrogen peroxide solution was applied for fifteen minutes. Sections or slides were then sequentially incubated with serum, primary antibody, and secondary antibody. Tyramide signal amplification working buffer (ABclonal, RK05905) was added for ten minutes, followed by washing with 1× phosphate-buffered solution. This cycle, from antigen retrieval to tyramide signal amplification buffer incubation, was repeated until all target proteins were labeled. Additionally, TUNEL staining was conducted according to the protocol of a commercial kit (Beyotime, C1088). Immunofluorescence images were captured using a fluorescence microscope, and co-localized dots were analyzed using ImageJ software.

### Confocal imaging

The sections or slides were mounted onto a confocal dish and examined using a confocal imaging system (Leica, Switzerland). Based on the co-localized regions in the immunofluorescence images, the co-localization was enhanced and captured in the RGB channel. The confocal images were merged and analyzed using ImageJ software, with its Colocalization Finder plugin utilized to calculate the Pearson’s correlation coefficient (Rr value). This coefficient ranged from −1.0 to 1.0, where negative values indicated negative correlation, positive values indicated positive correlation, and the strength of correlation increased with the absolute value of Rr [33].

### The prediction for protein-protein interactions (PPIs)

The prediction of protein-protein interactions (PPIs) relied on data obtained from the mass spectrum analysis within the BioGRID database [34]. The inference of PINK1-MIF interaction occurred when other proteins acted as bait, being captured from cell extracts using either polyclonal antibodies or epitope tags, and the associated interacting partner was identified through mass spectrometric techniques. The visuals depicting PPIs were retrieved from the BioGRID website.

### Co-immunoprecipitation

The magnetic beads coated with protein A + G (Beyotime, P2179S) were placed on a rotary mixer along with the target proteins or IgG antibody at room temperature for 1 h. This step facilitated the formation of a complex, where the antibody bound to the magnetic beads. Subsequently, the lysate containing the protein sample was added to the complex and left to incubate overnight at four degrees Celsius. Proteins capable of interacting with the target proteins were pulled down together. The complex was then washed and incubated with 1× sodium dodecyl sulfate-polyacrylamide gel electrophoresis (SDS-PAGE) loading buffer at one hundred degrees Celsius for ten minutes. The supernatant was collected for subsequent experiments. The type and expression of the pulled-down proteins were identified through immunoblotting.

### Immunoblotting

The protein samples obtained from HK-2 cells or renal tissues were denatured and separated using 10%, 12%, and 15% SDS-PAGE. Subsequently, the blots were transferred onto polyvinylidene difluoride membranes and incubated with primary antibodies overnight at four degrees Celsius. Horseradish peroxidase-labeled secondary antibodies were employed to label the protein blots. Immunoblotting images were scanned and analyzed using Image J software. The densitometry of target proteins was normalized against GAPDH or β-Actin.

### JC-1 and apoptosis staining

HK-2 cells were seeded into two twelve-well plates based on the assigned groups. Once the cell density reached 80%, the designated pretreatment drugs and LPS were added to the HK-2 cells for incubation. The incubation was halted at various time intervals, and one plate of HK-2 cells was detached using trypsin solution. These cells were then subjected to staining with JC-1 (MedChemExpress, HY-K0601) at thirty-seven degrees Celsius for twenty minutes, followed by washing with 1× phosphate-buffered solution. Subsequently, the JC-1 stained HK-2 cells were observed under a fluorescence microscope and quantified using flow cytometry. Similarly, HK-2 cells were exposed to annexin V-FITC and PI (MedChemExpress, HY-K1073) at room temperature for twenty minutes to identify apoptotic cells, which were then enumerated using flow cytometry (FCM).

### Transmission electron microscope (TEM)

The renal tissue or HK-2 cell samples were initially fixed using 2.5% glutaraldehyde. After washing, they were re-fixed with osmium tetroxide solution and then sliced into nanoscale sections. These slices were subsequently stained with uranium acetate and lead citrate before being washed again. The morphology of organelles was observed and captured under magnifications ranging from 5000x to 10,000x using the TEM imaging system (Tecnai, USA). Pathologists identified and marked pathological characteristic structures such as damaged mitochondria, autophagosomes, and mitophagosomes.

### Quantitative polymerase chain reaction (qPCR)

The RNA from transfected HK-2 cells was isolated using a commercially available kit (Vazyme, RC112-01). Subsequently, complementary DNA (cDNA) was synthesized via reverse transcription (Vazyme, R423-01). The cDNA was then combined with primers, and the silencing effect of siRNA was assessed using quantitative PCR (qPCR). All primers were procured from Tsingke Biotech Corporation (Beijing, China). The primer sequences for human *PINK1* were as follows: forward, 5′-GGGAGTATGGAGCAGTCACTTAC-3′, reverse, 5′-GCAGGGTACAGGGATAGTTCTTC-3′. The primer sequences for human *PRKN* were: forward, 5′-GGCTGTCCCAACTCCTTGATTAA-3′, reverse, 5′-GCTTCTTTACATTCCCGGCAGAA-3′. The primer sequences for human *Actin* were: forward, 5′-CCTTCCTGGGCATGGAGTC-3′, reverse, 5′-TGATCTTCATTGTGCTGGGTG-3′.

### Correlation analysis

The correlation in different groups was analyzed by the software GraphPad Prism 10. All data were satisfied with the Gaussian distribution. The strength of the correlation was presented with the R-value of Pearson’s correlation coefficient (negative correlation: −1.0 to 0, positive correlation: 0–1.0, the correlation strength increased with the absolute value of R). The equation and the *P* value were calculated according to the Simple Linear Regression.

### Seven-day survival curve

Survival curves spanning 7 days were generated using GraphPad Prism 10 software based on the count of surviving mice following CLP surgery. The *P* value associated with the survival curves was determined using both the Log-Rank Test and the *Gehan*–*Breslow*–*Wilcoxon* Test.

### Statistical analysis

Statistical analysis was conducted using GraphPad Prism 10 software. Qualitative data were presented as means ± standard error of the mean (SEM). A two-tailed unpaired Student’s *t*-test was employed to compare two groups, while one-way analysis of variance (ANOVA) followed by *Tukey’s* post-tests was utilized for multiple-group comparisons involving one independent factor. For comparisons involving multiple independent factors, two-way ANOVA followed by *Tukey’s* post-tests was applied. A *P*-value below 0.05 was deemed indicative of a significant difference.

## Results

### LPS-induced the upregulation of intracellular MIF was positively correlated with mitophagy

**Fig. 1:**
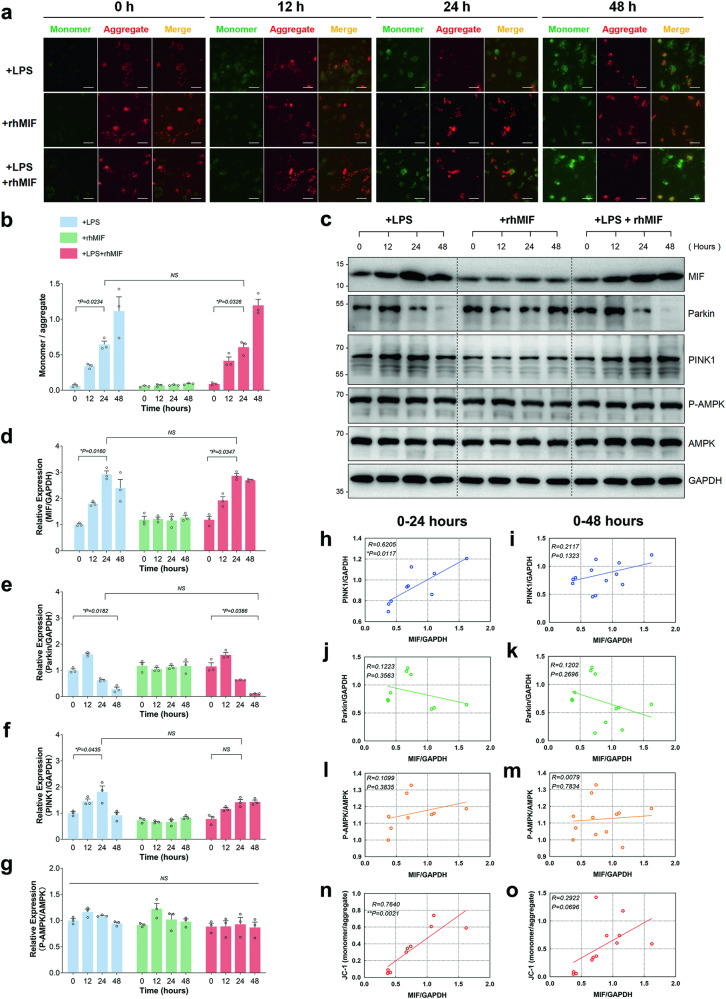
A positive correlation between the upregulation of intracellular MIF induced by LPS and mitophagy. **a** The images from the green channel (representing monomer) and red channel (representing aggregate) were combined. Scale bar : 50 μm. **b** The depolarization of ΔΨm was assessed by counting the dots in JC-1 stained HK-2 cells, indicating the ratio of monomer/aggregate. **c** The expressions of MIF, PINK1, Parkin, AMPK, P-AMPK, and GAPDH were evaluated by immunoblotting at four time points (0, 12, 24, 48 h) during LPS stimulation. The relative expression of these proteins was presented as ratios: **d** MIF/GAPDH, **e** Parkin/GAPDH, **f** PINK1/GAPDH, and **g** P-AMPK/AMPK. Correlation analyses were conducted for the expression of MIF/GAPDH with (**h**, **i**) PINK1/GAPDH, (**j**, **k**) Parkin/GAPDH, (**l**, **m**) P-AMPK/AMPK, and (**n**, **o**) JC-1 (monomer/aggregate) during 0–24 h and 0–48 h of LPS stimulation. The goodness of fit was expressed as an *R*-value, with negative correlation ranging from −1.0 to 0 and positive correlation from 0 to 1.0. Data were presented as mean ± SEM, with *n* = 3. Statistical significance was denoted as *P < 0.05, **P < 0.01, ***P < 0.001, and ****P < 0.0001.

Mitochondrial membrane potential (ΔΨm) depolarization serves as a crucial indicator of mitochondrial damage and marks the onset of mitophagy in normal cells. HK-2 cells exposed to LPS were categorized into four groups based on different time intervals (0, 12, 24, and 48 h). Furthermore, to assess the impact of exogenous MIF, another group of HK-2 cells was treated with rhMIF. The monomer/aggregate ratio increased by approximately 9.92-fold after 24 h of LPS stimulation or LPS combined with rhMIF treatment. Notably, rhMIF did not alter this upward trend when compared between the LPS-only and LPS with rhMIF groups. Similar trends were observed with no apparent change in the monomer/aggregate ratio in the rhMIF-treated group alone (Fig. 1a, b). Concurrently, MIF expression levels increased over time with LPS stimulation, both in the LPS-only and LPS with rhMIF groups, whereas no significant change was noted in the rhMIF-treated group alone (Fig. 1c, d). A similar increasing pattern was observed in PINK1 expression, although no significant difference was observed at 24 h in the LPS with rhMIF group (Fig. 1c, f). Parkin expression showed a slight upregulation at 12 h (non-significant) and then declined between 24 and 48 h in both the LPS-only and LPS with rhMIF groups (Fig. 1c, e). Notably, rhMIF did not influence Parkin expression when comparing between the LPS-only and LPS with rhMIF groups, nor within the rhMIF-treated group alone. Additionally, there were no discernible changes in the P-AMPK/AMPK ratio (indicative of AMPK pathway activation) across all groups (Fig. 1c, g). Thus, the PINK1-Parkin pathway was initially activated and subsequently suppressed under LPS stimulation, a phenomenon seemingly independent of the AMPK pathway and exogenous MIF during the 0–48 h period.

To investigate the association between MIF and mitophagy, we examined the relationship between the relative expression of MIF/GAPDH and other ratios during 24 h or 48 h of LPS stimulation. The relative expression of MIF/GAPDH showed a positive correlation with the relative expression of PINK1/GAPDH (Fig. 1h, *R* = 0.6205, *P* = 0.0117), as well as the JC-1 ratio (Fig. 1n, *R* = 0.7640, *P* = 0.0021). However, no significant correlation was observed between the relative expression of MIF and Parkin (Fig. 1j), or MIF and P-AMPK (Fig. 1l). Likewise, there was no correlation between them during the 0–48 h period (Fig. 1i, k, m, o).

### Knockdown of MIF reduced LPS-induced mitochondria damage through activating the PINK1-Parkin pathway of mitophagy

**Fig. 2:**
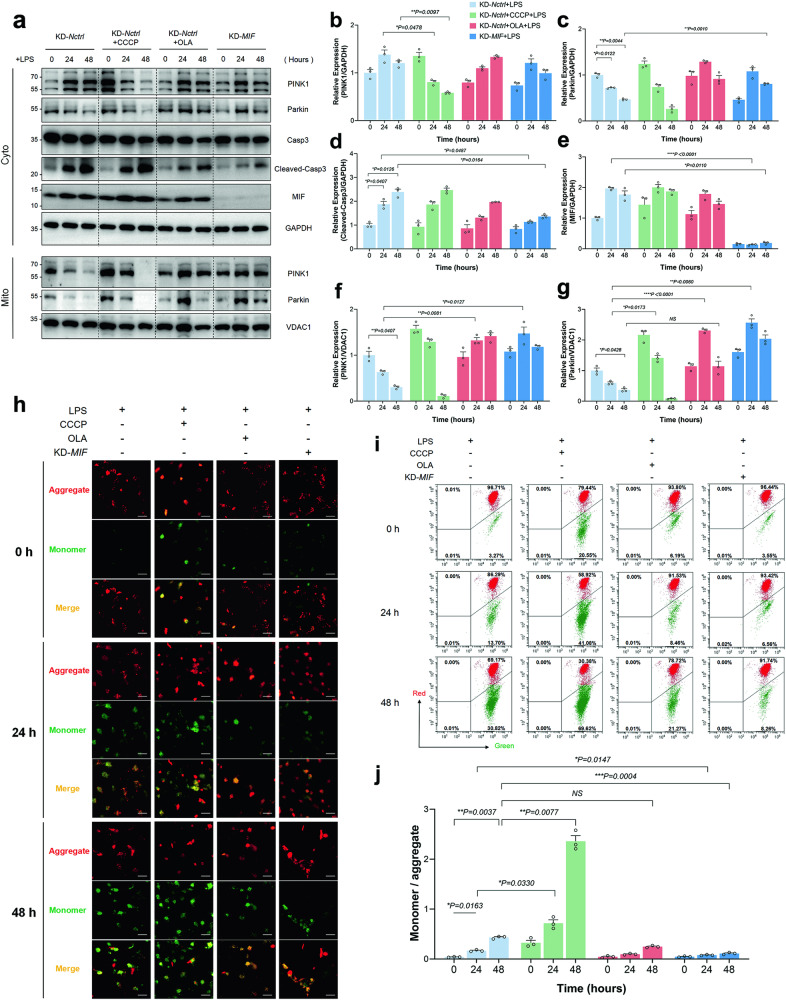
Knockdown of MIF reduces LPS-induced mitochondria damage through activating the PINK1-Parkin pathway of mitophagy. **a** The levels of MIF, caspase3 (Casp3), cleaved-Casp3, PINK1, Parkin, and GAPDH in the cytoplasmic fraction (Cyto), as well as PINK1, Parkin, VDAC1 in the mitochondrial enriched fraction (Mito), were assessed using immunoblotting. The relative expression of these proteins was represented as ratios: **b** PINK1/GAPDH, **c** Parkin/GAPDH, **d** cleaved-Casp3/GAPDH, **e** MIF/GAPDH, **f** PINK1/VDAC1, and **g** Parkin/VDAC1. **h** Representative images of JC-1 stained HK-2 cells were provided, with the green channel (monomer) and red channel (aggregate) merged, and a scale bar of 50 μm included. **i** The number of JC-1 stained HK-2 cells was quantified by FCM in both the green and red channels. **j** The depolarization of ΔΨm was evaluated based on the ratio of monomer/aggregate. Data were presented as mean ± SEM, with *n* = 3. Statistical significance was indicated as *P < 0.05, **P < 0.01, ***P < 0.001, and ****P < 0.0001.

Mitophagy induction in HK-2 cells was initiated using CCCP (10 μM, administered 2 h prior) or OLA (2 μM, administered 12 h prior). HK-2 cells in the KD-*MIF* group were transfected with knockdown lentivirus, resulting in a significant decrease in MIF expression as confirmed by immunoblotting (Fig. 2a, e). CCCP pretreatment was administered to HK-2 cells in the KD-*Nctrl* + CCCP + LPS group, while OLA was administered to those in the KD-*Nctrl* + OLA + LPS group. Samples were harvested at each designated time point (0, 24, and 48 h). It was observed that both mitochondrial and cytoplasmic PINK1 levels were rapidly depleted by LPS + CCCP (Fig. 2a, b, f), mirroring the trend observed for Parkin expression, which progressively decreased over time following LPS or LPS + CCCP stimulation (Fig. 2a, c, g). The variation in PINK1 expression within the KD-*Nctrl* + LPS group was not clearly evident in the cytoplasmic fraction, unlike Parkin (Fig. 2a–c). OLA induced mitophagy with less intensity compared to CCCP, and even enhanced the recruitment of both PINK1 and Parkin to mitochondria during LPS stimulation (Fig. 2a, f, g). However, by 48 h, a significant reduction in Parkin levels in the mitochondrial enriched fraction was observed, resulting in similar Parkin expression between the KD-*Nctrl* + LPS and KD-*Nctrl* + OLA + LPS groups (Fig. 2a, g). Conversely, knockdown of MIF led to substantial increases in both PINK1 and Parkin expression at 24 h of LPS stimulation, and maintained high Parkin expression at 48 h (KD-*MIF* + LPS vs KD-*Nctrl* + LPS, Fig. 2a, f, g). Similarly, the apoptosis core protein cleaved-casp3 increased over time following LPS or LPS + CCCP challenge, a trend alleviated by MIF knockdown but not by OLA (Fig. 2a, d). Furthermore, we observed an increase in the ratio of monomer/aggregate with LPS stimulation over time, particularly pronounced in the KD-*Nctrl* + CCCP + LPS group (mitophagy exhaustion). OLA slightly reduced the ratio at 24 h, while did not reduce the ratio at 48 h. However, knockdown of MIF decreased the ratio at both time points (Fig. 2h–j).

### MIF over-expression (OE-*MIF*) further aggravated LPS injury through suppressing the PINK1-Parkin pathway of mitophagy, which could be reversed by ISO-1

**Fig. 3:**
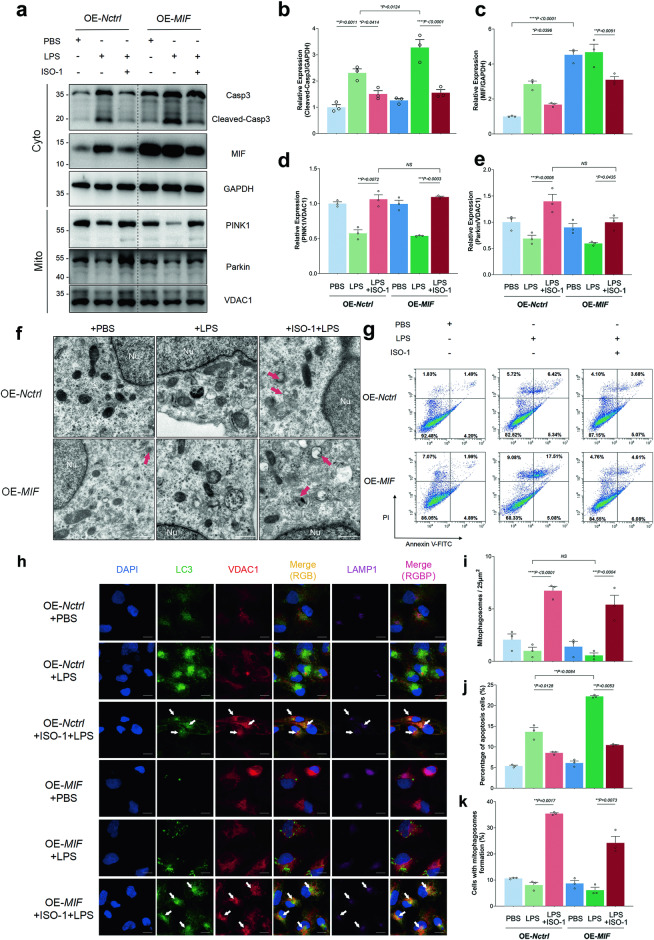
The exacerbation of LPS-induced injury due to MIF over-expression (OE-MIF) is attributed to the inhibition of the PINK1-Parkin pathway of mitophagy, a phenomenon alleviated by ISO-1. **a** The levels of MIF, caspase3 (Casp3), cleaved-Casp3, and GAPDH in the cytoplasmic fraction (Cyto), as well as PINK1, Parkin, and VDAC1 in the mitochondrial enriched fraction (Mito), were quantified using immunoblotting. The relative expression of these proteins was indicated as ratios: **b** cleaved-Casp3/GAPDH, **c** MIF/GAPDH, **d** PINK1/VDAC1, and **e** Parkin/VDAC1. **f** TEM images were provided to illustrate the morphology of mitophagosomes, highlighted by red arrows, with a scale bar of 500 nm. **g** Apoptotic HK-2 cells stained with annexin V and PI were counted using FCM. **h** Representative images of triple-labeling immunofluorescence in different HK-2 cell groups were presented, showing the colocalization of LC3 (green), VDAC1 (red), and LAMP1 (purple), marking the mitophagosomes by a white arrow, with a scale bar of 10 μm. **i** The average number of mitophagosomes per 25 μm^2^ was calculated in ten random TEM fields. **j** The percentage of apoptotic cells was determined by FCM. **k** The presence of HK-2 cells with mitophagosome formation was evaluated based on the triple-labeling immunofluorescence images. Data were expressed as mean ± SEM, with n = 3. Statistical significance was indicated as *P < 0.05, **P < 0.01, ***P < 0.001, and ****P < 0.0001.

HK-2 cells were subjected to MIF over-expression (OE-*MIF*) through lentiviral transfection, while ISO-1 was utilized to inhibit MIF’s biological activity. Cells in the OE-*Nctrl* + ISO-1 + LPS and OE-*MIF* + ISO-1 + LPS groups were pre-treated with ISO-1 (20 μM) 4 h prior, followed by LPS challenge for 24 h. MIF expression significantly increased in the OE-*MIF* group (Fig. 3a, c). OE-*MIF* further heightened cleaved-casp3 expression in the cytoplasm, a effect reversed by ISO-1. Concurrently, ISO-1 also attenuated cleaved-casp3 expression in the OE-*Nctrl* + ISO-1 + LPS group compared to the OE-*Nctrl* + LPS group (Fig. 3a, b). This trend mirrored MIF expression (OE-*Nctrl* + ISO-1 + LPS group vs OE-*Nctrl* + LPS group, Fig. 3a, c). Additionally, ISO-1 mitigated LPS’s suppressive effect, enhancing PINK1 and Parkin recruitment to mitochondria (OE-*Nctrl* + ISO-1 + LPS group vs OE-*Nctrl* + LPS group or OE-*MIF* + ISO-1 + LPS group vs OE-*MIF* + LPS group, Fig. 3a, d, e). We observed an increase in mitophagosome count in the OE-*Nctrl* + ISO-1 + LPS and OE-*MIF* + ISO-1 + LPS groups (vs OE-*Nctrl* + LPS group or OE-*MIF* + LPS group respectively, Fig. 3f, i). Approximately 3.55 times more HK-2 cells exhibited mitophagosome formation in the OE-*Nctrl* + ISO-1 + LPS group compared to the OE-*Nctrl* + LPS group, and 2.32 times more in the OE-*MIF* + ISO-1 + LPS group compared to the OE-*MIF* + LPS group (Fig. 3h, k). LPS induced apoptosis in HK-2 cells, exacerbated by OE-*MIF* but mitigated by ISO-1 (Fig. 3g, j).

### Increasing MIF restrained mitophagy through disturbing the interaction of PINK1 and Parkin

**Fig. 4:**
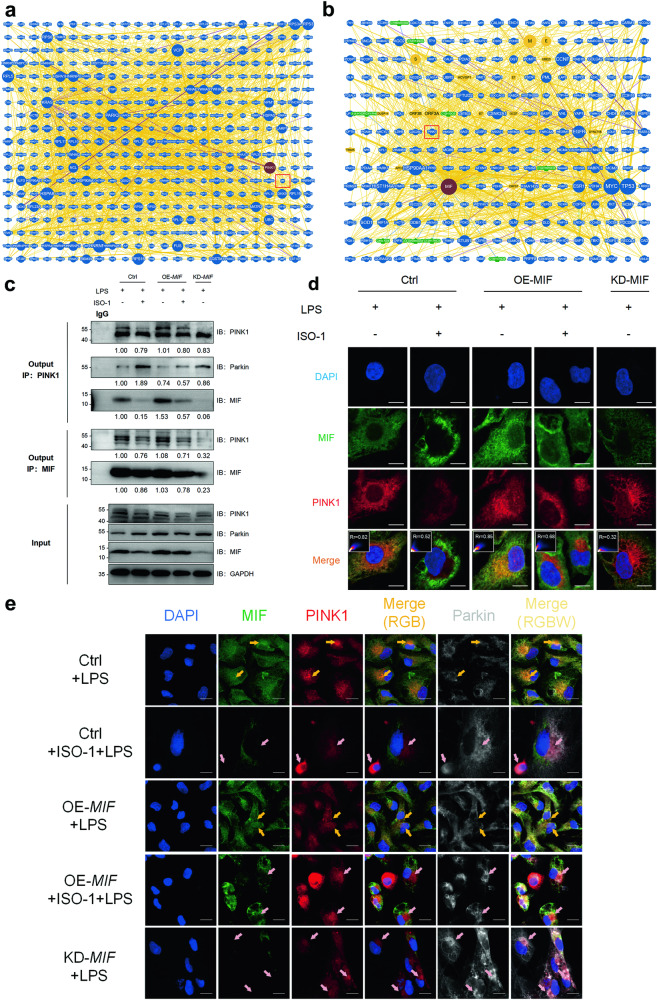
Increasing MIF restrained mitophagy through disturbing the interaction of PINK1 and Parkin. **a** The PPIs of PINK1 (MIF was marked by red box). **b** The PPIs of MIF (PINK1 was marked by red box) **c** The protein interaction of PINK1-MIF and PINK1-Parkin was measured by co-immunoprecipitation and immunoblotting. The gray value ratios were listed below each blot, reflecting the degree of interaction. **d** Representative confocal images showed the colocalization of MIF (green) and PINK1 (red) in different groups of HK-2 cells. Scale bar: 5 μm. The correlation of colocalization was presented as the Rr value of *Pearson’s* correlation coefficient (negative correlation: −1.0 to 0, positive correlation: 0–1.0, the correlation strength increased with the absolute value of Rr). **e** Representative images of triple-labeling MIF (green), PINK1 (red), and Parkin (white) immunofluorescence showed the colocalization of MIF & PINK1 (yellow arrows) and PINK1 & Parkin (pink arrows). Scale bar: 10 μm. *n* = 3. *P <0.05, **P < 0.01, ***P < 0.001, ****P < 0.0001.

The PPIs network within the BioGRID database anticipated the interaction linking MIF and PINK1 (Fig. 4a, b). Utilizing co-immunoprecipitation, we verified this interaction, observing that both MIF and PINK1 proteins could be precipitated by the respective antibodies of each other (Fig. 4c). Under conditions of LPS stimulation or overexpression of MIF (OE-*MIF*), the binding between these two proteins was notably strengthened compared to treatment with ISO-1 or under conditions of MIF knockdown (KD-*MIF*), as confirmed by immunoblotting and confocal imaging results (Fig. 4c). Confocal images further indicated increased colocalization of MIF-PINK1 in the Ctrl+LPS (Rr=0.82) and OE-*MIF* + LPS (Rr = 0.85) groups, while decreased colocalization was observed in the Ctrl+ISO-1 + LPS (Rr = 0.52), OE-*MIF* + ISO-1 + LPS (Rr = 0.68), and KD-*MIF* + LPS (Rr = 0.32) groups (Fig. 4d). Triple-labeling immunofluorescence images demonstrated that higher colocalization between PINK1 and Parkin occurred when the interaction between PINK1 and MIF was diminished, consistent with immunoblotting findings (Fig. 4c, e).

### Knockdown of MIF and T0467 both improved LPS injury through recruiting Parkin to mitochondria

**Fig. 5:**
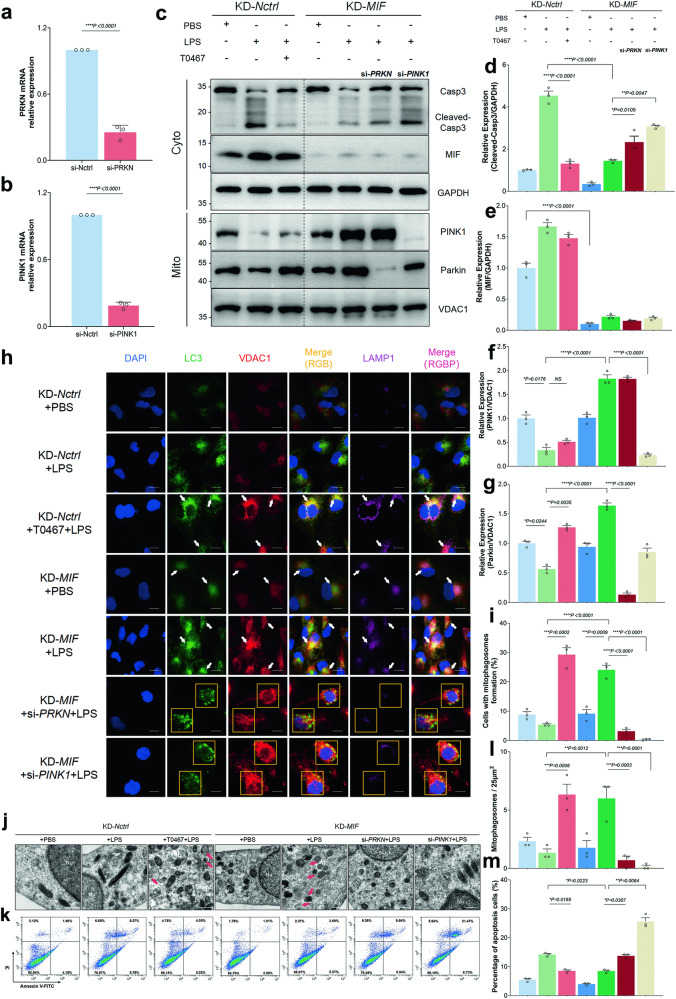
MIF knockdown and T0467 administration both enhanced protection against LPS-induced injury by facilitating the recruitment of Parkin to mitochondria. The qPCR was utilized to assess the relative expression levels of (**a**) *PRKN* mRNA and (**b**) *PINK1* mRNA, comparing the si-*Nctrl* group with the si-*PRKN* or si-*PINK1* group. Additionally, **c** the levels of MIF, Casp3, cleaved-Casp3, and GAPDH in the cytoplasmic fraction (Cyto), as well as PINK1, Parkin, and VDAC1 in the mitochondrial enriched fraction (Mito), were determined via immunoblotting. The relative expression ratios of these proteins were quantified: **d** cleaved-Casp3/GAPDH, **e** MIF/GAPDH, **f** PINK1/VDAC1, and **g** Parkin/VDAC1. **h** Representative images from triple-labeling immunofluorescence of various HK-2 cell groups were analyzed. The potential mitophagosomes, based on the colocalization of LC3 (green), VDAC1 (red), and LAMP1 (purple), were identified by white arrows, while areas without colocalization were presented within yellow boxes. Scale bar: 10 μm. **i** The presence of mitophagosome formation in HK-2 cells was assessed using triple-labeling immunofluorescence images. **j** Representative TEM images depicted the morphology of mitophagosomes (mitochondria-like structures enclosed by autophagosomes, marked by red arrows). Scale bar: 500 nm. **k** Apoptosis in HK-2 cells, stained with annexin V-FITC and PI, was quantified using FCM. **l** The mean number of mitophagosomes per 25 μm^2^ was calculated from ten random fields of TEM view. **m** The percentage of apoptotic cells was determined. Data were presented as mean ± SEM. *n* = 3. *P < 0.05, **P < 0.01, ***P < 0.001, ****P < 0.0001.

Considering the mechanism of MIF, we identified a novel mitophagy inducer, T0467, aimed at alleviating the suppression of the PINK1-Parkin pathway. T0467 enhanced PINK1’s ability to recruit Parkin to mitochondria, even under PINK1 inhibition. Before LPS stimulation, the transcription of *PINK1* and *PRKN* genes was silenced using siRNA. Significant reductions in *PINK1* and *PRKN* mRNA expression were observed in the si-*PINK1* or si-*PRKN* groups compared to the si-*Nctrl* group (Fig. 5a, b). Moreover, siRNA decreased the recruitment of PINK1 or Parkin to mitochondria and suppressed mitophagosome formation in the KD-*MIF*+si-*PINK1* + LPS or KD-*MIF*+si-*PRKN* + LPS groups compared to the KD-*MIF* + LPS group (Fig. 5c, f, g, h, i). Both T0467 and KD-*MIF* reduced LPS-induced up-regulation of cleaved-casp3 and increased the recruitment of Parkin to mitochondria (KD-*Nctrl* + T0467 + LPS group vs KD-*Nctrl* + LPS group, and KD-*MIF* + LPS group vs KD-*Nctrl* + LPS group, Fig. 5c, d, f, g). Importantly, T0467’s effect was unrelated to MIF expression changes (Fig. 5c, e). T0467 promoted approximately 5.38-times more HK-2 cells exhibiting mitophagosome formation (KD-*Nctrl* + T0467 + LPS group vs KD-*Nctrl* + LPS group), while KD-*MIF* increased it by 4.43 times (KD-*MIF* + LPS group vs KD-*Nctrl* + LPS group, Fig. 5h, i). Similarly, both T0467 and KD-*MIF* increased mitophagosome count and reduced the number of apoptotic cells (Fig. 5j–m).

### ISO-1 and T0467 both improved sepsis-associated RTECs injury through activating PINK1-Parkin pathway of mitophagy

**Fig. 6:**
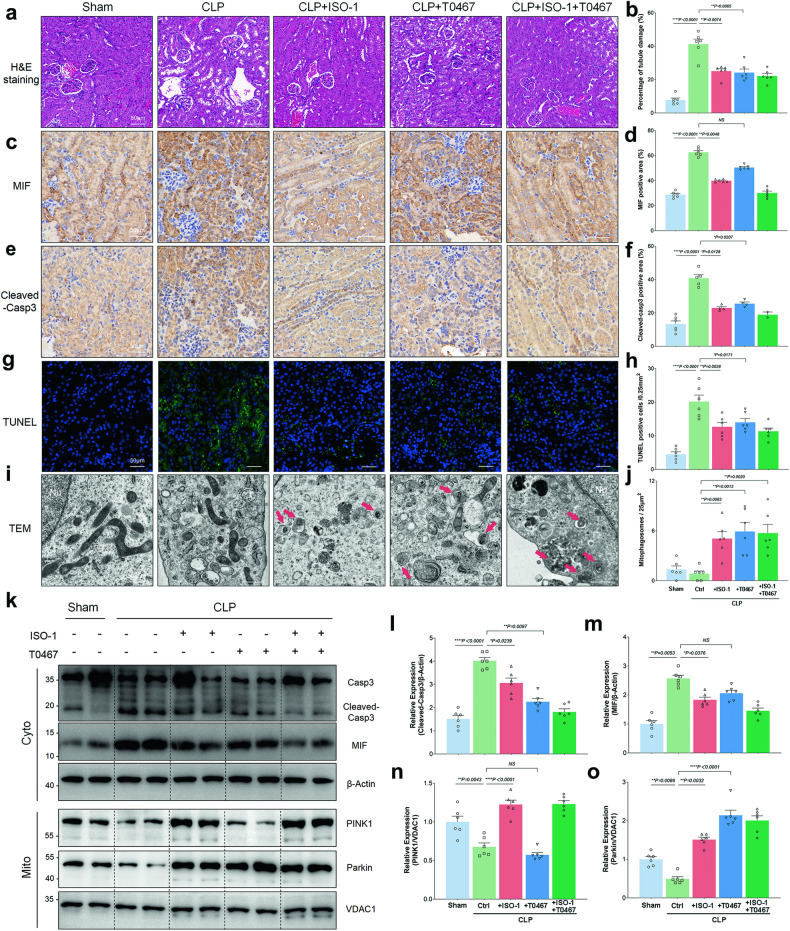
ISO-1 and T0467 both ameliorated RTEC injury induced by CLP through enhancing the PINK1-Parkin pathway of mitophagy. **a**, **b** Hematoxylin and eosin (H&E) staining was performed on renal tissues, and representative images were obtained to evaluate the percentage of tubule damage in each group. Scale bar: 50 μm. (**c**, **d**) Immunohistochemical staining using MIF antibody was conducted on renal tissues, and representative images were captured to calculate the percentage of the area stained with MIF in different groups. Scale bar: 50 μm. **e**, **f** Immunohistochemical staining with cleaved-casp3 antibody was performed on renal tissues, and representative images were acquired to determine the percentage of the area stained with cleaved-casp3 in different groups. Scale bar: 50 μm. **g**, **h** Renal tissues were subjected to immunofluorescence staining with TUNEL, and representative images were taken to quantify the number of TUNEL-positive cells per 0.25 mm^2^ in different groups. Scale bar: 50 μm. **i** Representative TEM images displayed the morphology of mitophagosomes (mitochondria-like structures enclosed by autophagosomes, indicated by red arrows). Scale bar: 500 nm. **j** The mean number of mitophagosomes per 25 μm^2^ was calculated from ten random fields observed in TEM view. **k** Immunoblotting was utilized to measure the expression levels of MIF, Casp3, cleaved-Casp3, and β-Actin in the cytoplasmic fraction (Cyto), as well as PINK1, Parkin, and VDAC1 in the mitochondrial enriched fraction (Mito). The relative expression ratios of these proteins were quantified: **l** cleaved-Casp3/β-Actin, **m** MIF/β-Actin, **n** PINK1/VDAC1, and **o** Parkin/VDAC1. Data were presented as mean ± SEM. *n* = 6. *P < 0.05, **P < 0.01, ***P < 0.001, ****P < 0.0001.

The therapeutic efficacy of ISO-1 and T0467 was evaluated in vivo. Mice in the CLP + ISO-1 group and CLP + T0467 + ISO-1 group received ISO-1 treatment (30 mg/kg mouse weight), while mice in the CLP + T0467 group and CLP + T0467 + ISO-1 group were treated with T0467 (5 mg/kg mouse weight). H&E staining images revealed approximately 21.21% reduction in tubular damage area in the CLP + ISO-1 group and 27.08% in the CLP + T0467 group compared to the CLP (CLP+Ctrl) group (Fig. 6a, b). Serial sections of renal tissues demonstrated a consistent high similarity in the expression and localization of MIF and cleaved-casp3. Immunohistochemical images indicated that both ISO-1 and T0467 reduced cleaved-casp3 expression in renal tissues (Fig. 6c–f). Similar findings were observed in TUNEL staining images and cleaved-casp3 expression measured by immunoblotting (Fig. 6g, h, k, l). Conversely, there was an approximate 5.96-fold increase in mitophagosomes in the CLP + ISO-1 group and 6.96-fold in the CLP + T0467 group compared to the CLP group (Fig. 6i, j). Both PINK1 and Parkin expression in mitochondria were elevated, with only Parkin increasing in the CLP + T0467 group (Fig. 6k, n, o). Furthermore, we found that T0467’s effect in activating the PINK1-Parkin pathway was independent of MIF expression (CLP + T0467 group vs CLP group, Fig. 6k, m). Additionally, the combined application of ISO-1 and T0467 did not result in an enhanced effect on activating mitophagy and improving RTECs injury (CLP + ISO-1 + T0467 group vs CLP + ISO-1/CLP + T0467 group, Fig. 6a, b, g, h, k, l, o).

In order to eliminate the confounding factors, murine primary tubular epithelial cells (mPTECs) were isolated for experimentation. Our findings indicate that both ISO-1 and T0467 exhibited similar anti-injury properties in vitro by facilitating the translocation of PINK1 and Parkin to mitochondria, enhancing mitophagosome formation, and reducing apoptosis in mPTECs (The supplementary figure, Fig. S6). Therefore, ISO-1 and T0467 were effective in mitigating sepsis-induced RTECs injury by activating the PINK1-Parkin pathway of mitophagy.

### ISO-1 and T0467 motivated the interaction of PINK1 and Parkin and further improved renal function in SA-AKI

**Fig. 7:**
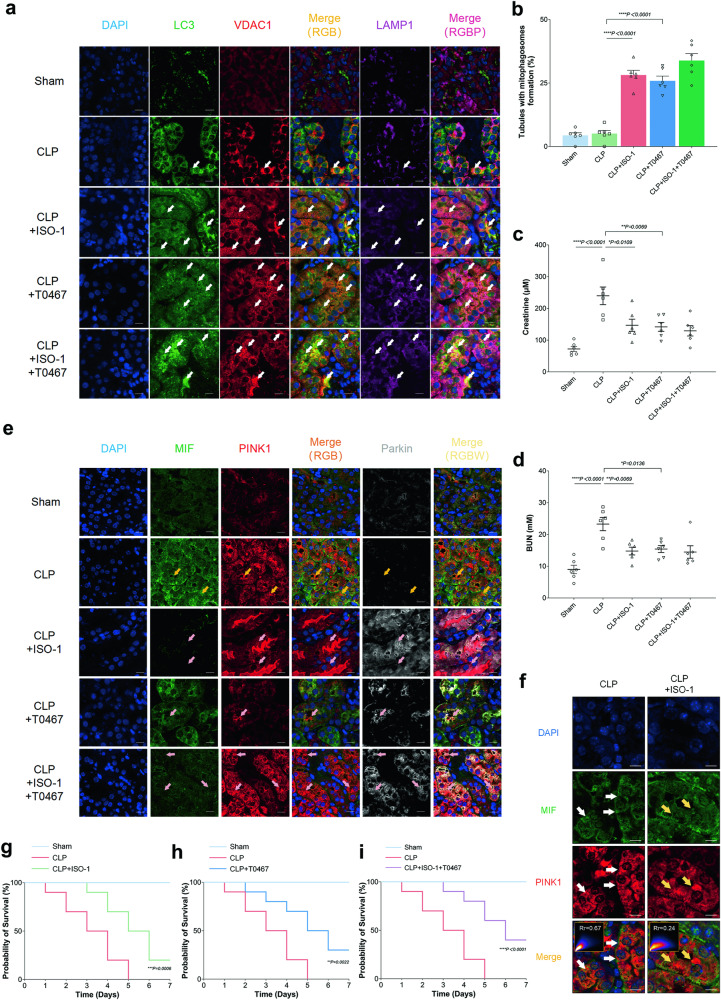
ISO-1 and T0467 facilitated the interaction between PINK1 and Parkin, thereby enhancing renal function in SA-AKI. **a** Representative images from triple-labeling immunofluorescence in various groups. Mitophagosomes were indicated by white arrows, based on the colocalization of LC3 (green), VDAC1 (red), and LAMP1 (purple). Scale bar: 20 μm. **b** Evaluation of tubules with mitophagosome formation in the triple-labeling immunofluorescence images. **c** Creatinine levels. **d** Blood urea nitrogen (BUN) levels. **e** Representative images from triple-labeling immunofluorescence showing potential colocalization of MIF (green) & PINK1 (red), and PINK1 & Parkin (white). Colocalization of MIF and PINK1 was highlighted by yellow arrows. Colocalization of PINK1 and Parkin was marked by pink arrows. Scale bar: 20 μm. **f** Representative confocal images depicting the colocalization of MIF (green) and PINK1 (red) in the CLP and CLP + ISO-1 groups. The white arrows denoted the likely colocalization of MIF and PINK1, in contrast to the yellow arrows. Scale bar: 10 μm. The correlation of colocalization was represented by the Rr value of Pearson’s correlation coefficient (negative correlation: −1.0 to 0, positive correlation: 0–1.0; correlation strength increased with the absolute value of Rr). Seven-day survival curves of mice: **g** comparison of CLP and CLP + ISO-1 groups, **h** comparison of CLP and CLP + T0467 groups, **i** comparison of CLP and CLP + ISO-1 + T0467 groups. Data were presented as mean ± SEM. *n* = 6. *P < 0.05, **P < 0.01, ***P < 0.001, ****P < 0.0001.

In our subsequent investigation, we examined the co-localization of mitophagosomes (LC3-VDAC1-LAMP1). Immunofluorescence images revealed a 5.54-fold increase in tubules exhibiting mitophagosome formation in the CLP + ISO-1 group and a 5.03-fold increase in the CLP + T0467 group compared to the CLP group (Fig. 7a, b). Notably, plasma creatinine levels were significantly reduced to 146.7 μM in the CLP + ISO-1 group and 141.8 μM in the CLP + T0467 group, in contrast to 239.6 μM in the CLP group (Fig. 7c). Similarly, plasma BUN levels dropped to 14.76 mM in the CLP + ISO-1 group and 15.40 mM in the CLP + T0467 group, compared to 23.27 mM in the CLP group (Fig. 7d). Immunofluorescence images demonstrated two distinct co-localization areas, MIF-PINK1 and PINK1-Parkin, which were mutually exclusive, particularly in the CLP and CLP + ISO-1 groups. CLP induced the interaction between MIF and PINK1, a phenomenon reversed by ISO-1. Both ISO-1 and T0467 facilitated the interaction between PINK1 and Parkin, but T0467 less inhibited the MIF-PINK1 interaction (Fig. 7e). Confocal images indicated greater co-localization area in the CLP group (Rr = 0.67) and lesser in the CLP + ISO-1 group (Rr = 0.24, Fig. 7f). Moreover, both ISO-1 and T0467 improved the outcomes of CLP mice, as evidenced by the 7-day survival curves (Fig. 7g–i).

## Discussion

**Fig. 8:**
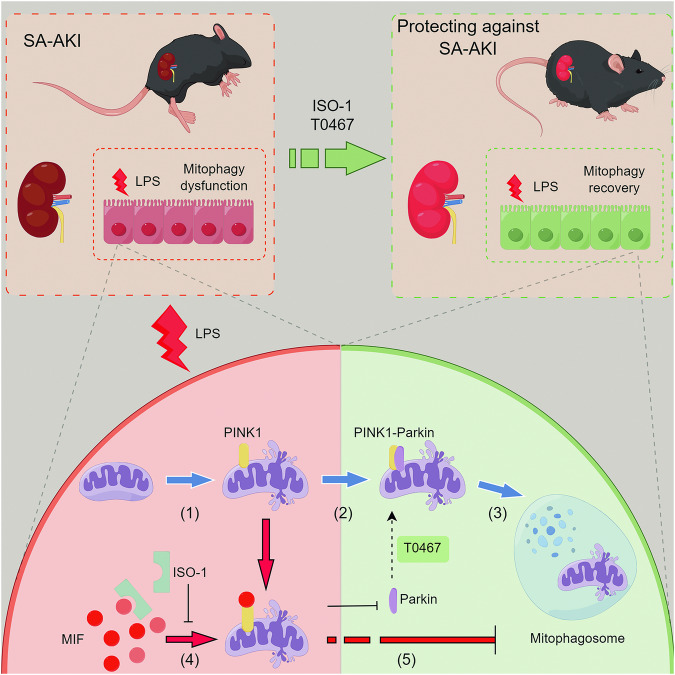
The schematic mechanism by which MIF inhibits mitophagy and leads to RTECs injury in SA-AKI by disrupting the protein interaction between PINK1 and Parkin. (1) LPS triggered mitochondrial damage. (2) Typically, PINK1 relocated to the impaired mitochondria and recruited Parkin. (3) Parkin interacted with PINK1 and facilitated the formation of mitophagosomes, leading to the degradation of damaged mitochondria. T0467 enhanced the recruitment of Parkin to mitochondria. (4) However, LPS induced the upregulation of MIF. Elevated MIF interacted with PINK1 before Parkin, a process reversible by ISO-1. (5) This interaction hindered the formation of mitophagosomes, resulting in RTECs injury in SA-AKI. Generally, ISO-1 and T0467 both mitigated RTECs injury and improved renal function in SA-AKI by alleviating the inhibitory effect of MIF on the PINK1-Parkin pathway of mitophagy (By Figdraw).

Dysfunction in mitophagy, leading to renal tubular epithelial cell (RTEC) injury, is a critical pathomechanism in sepsis-associated acute kidney injury (SA-AKI). However, the mechanism by which inflammation in SA-AKI regulates the mitophagy pathway remains unclear. Previous research has shown that serum levels of macrophage migration inhibitory factor (MIF) increase with the severity of SA-AKI and can predict its early occurrence, suggesting a potential involvement of MIF in the pathological process of SA-AKI [15]. Additionally, our earlier study demonstrated that downregulating MIF attenuates RTEC injury and preserves renal function in SA-AKI [17]. In this investigation, we observed an upregulation of the pro-inflammatory factor MIF in SA-AKI models, indicating its role in modulating mitophagy. Through in vitro and in vivo experiments, we aimed to elucidate how MIF regulates the PINK1-Parkin pathway of mitophagy and its contribution to RTEC injury aggravation in SA-AKI. Initially, we ruled out the influence of exogenous MIF and the AMPK pathway and confirmed a correlation between MIF and mitophagy. Subsequently, we identified an interaction between PINK1 and MIF, which hindered the recruitment of Parkin to mitochondria, resulting in mitophagy dysfunction and apoptosis. Furthermore, ISO-1 and T0467 were shown to alleviate the inhibitory effect of MIF on the PINK1-Parkin pathway of mitophagy, thereby improving renal function and outcomes in SA-AKI mice (Fig. 8).

Numerous prior research studies have regarded MIF as a co-factor of the NLRP3 inflammasome. Colocalization of MIF with NLRP3 has been observed in various inflammatory conditions [19, 35]. NLRP3 is triggered by bacterial components such as LPS and nigericin, with activation occurring due to the accumulation of damaged mitochondria producing ROS and oxidized mitochondrial DNA [36, 37]. Recent investigations have shown that PINK1 deficiency enhances the activation of the NLRP3 pathway by inhibiting Parkin-mediated mitophagy [24, 38]. Consequently, we considered the connection between MIF and mitophagy in our study.

Mitochondria play a crucial role in energy metabolism and the regulation of programmed cell death. Dysfunction in mitochondria is implicated in numerous diseases, yet the underlying reasons for mitochondrial dysfunction in SA-AKI remain unclear. While the physiological and pathological mechanisms of mitochondria have been gradually elucidated, the specific triggers for mitochondrial dysfunction in SA-AKI have not been fully understood. Typically, mitofusin-2 (Mfn2) is commonly assessed to evaluate mitochondrial function, but the expression of PINK1 and Parkin changes independently of Mfn2 in SA-AKI [5]. Our study revealed that despite high levels of PINK1 expression, the recruitment of Parkin to mitochondria was hindered by the upregulation of MIF (Fig. 2a, b). This occurred because damaged mitochondria made it challenging to recruit and degrade PINK1, leading to its accumulation in the cytoplasm. Consequently, even if mitophagy was initiated, RTEC injury persisted during SA-AKI progression. Furthermore, the group with si-*PINK1* exhibited a more significant increase in apoptotic cells compared to the si-*PRKN* group (Fig. 5k, m). We hypothesized that si-*PINK1* might inhibit both Parkin-mediated mitophagy and BNIP3-mediated mitophagy simultaneously. However, as there is limited evidence suggesting that BNIP3-mediated mitophagy plays a role in SA-AKI, we did not investigate it further in our study.

However, differing viewpoints persist. Xu et al. in 2014 observed that MIF deficiency led to a decrease in total Parkin expression, which was expected to increase in myoblasts exposed to phenylephrine for 48 h [39]. Similar findings were echoed in the study by Smith et al. in 2019, where they found that MIF deficiency resulted in the downregulation of total Parkin in mouse lung tissue, thereby improving resistance to influenza viral infection [40]. We think that total cellular Parkin levels may serve as an indicator of impending changes in mitophagy, while Parkin localization within mitochondria reflects the ongoing status. Assessing Parkin levels within mitochondria may offer more compelling evidence, as its translocation aids in reactive oxygen species (ROS) elimination and prevents cell death [41–43]. Another area of contention revolves around whether the AMPK pathway plays a role in modulating mitophagy in SA-AKI. Wang et al. discovered that MIF rescued smoke extract-induced myopathic anomalies by promoting AMPK activation, mitophagy, and lysosomal function [44]. However, both our findings and a recent study have shown that the AMPK pathway is not activated in SA-AKI models, at least not in the early stages [45]. The discrepancy in disease models may contribute to conflicting conclusions.

Several limitations persisted in this study. Firstly, due to constraints in project funding, MIF gene knockout mice were not utilized. The inclusion of these mice would have strengthened the investigation into the mechanism of MIF in CLP mice. Additionally, we only employed ISO-1 and T0467 at recommended concentrations to activate the PINK1-Parkin pathway of mitophagy. Further research is needed to establish a dose-response relationship more rigorously. Furthermore, the protein interaction between MIF and PINK1 in the molecular structure was not elucidated. Molecular biological techniques are required to confirm the binding sites, domains, and their interactions. Nevertheless, we remain confident that these shortcomings will be addressed in future studies.

In conclusion, this study offers compelling evidence indicating that MIF up-regulation suppresses the PINK1-Parkin pathway of mitophagy in SA-AKI. MIF interferes with the protein interaction between PINK1 and Parkin, hindering Parkin’s recruitment to mitochondria and leading to RTECs apoptosis. However, this inhibitory effect of MIF can be reversed by ISO-1 and T0467, thereby ameliorating renal function and outcomes in SA-AKI mice by enhancing mitophagy. Therefore, targeting the mechanism by which MIF regulates mitophagy may hold promise as a potential therapeutic strategy for treating SA-AKI.

### Supplementary information

Full and uncropped western blots

Supplementary figure (Figure S6)

## Data Availability

The original data will be provided by the corresponding author upon reasonable request.
