# Supplementary material for: Macrophage migration inhibitory factor (MIF) suppresses mitophagy through disturbing the protein interaction of PINK1-Parkin in sepsis-associated acute kidney injury
Source: Cell Death Dis. 2024 Jul 2;15(7):473. doi: 10.1038/s41419-024-06826-z (PMC11220046; doi:10.1038/s41419-024-06826-z)
Supplement: Supplementary file 1 — Full and uncropped western blots [file 41419_2024_6826_MOESM1_ESM.pdf]

Figure 1

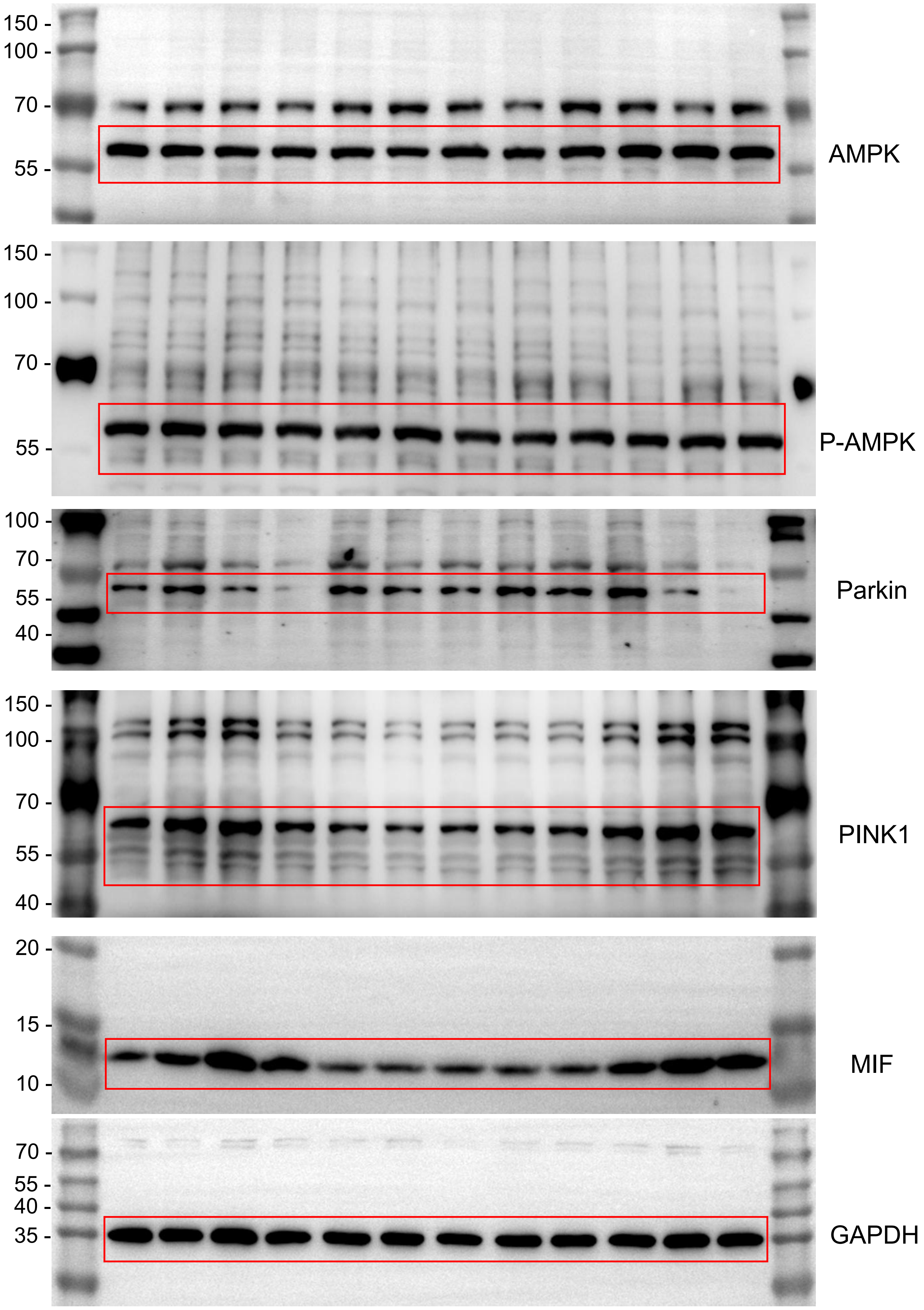
150 -
100 -
70 -
55 -
AMPK
150 -
100 -
70 -
55 -
P-AMPK
100 -
70 -
55 -
40 -
Parkin
150 -
100 -
70 -
55 -
40 -
PINK1
20 -
15 -
10 -
MIF
70 -
55 -
40 -
35 -
GAPDH

Figure 2

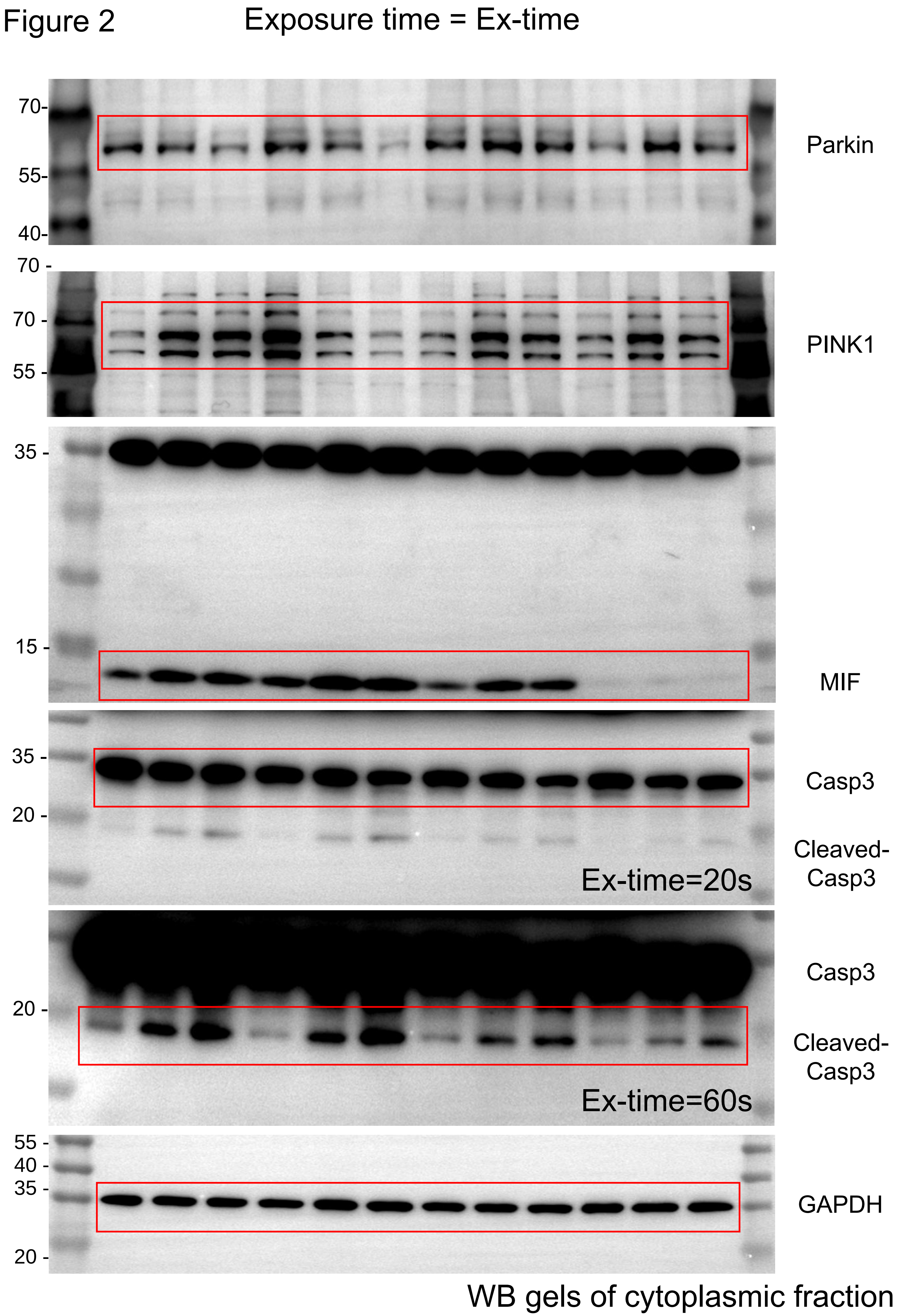
Exposure time = Ex-time
70-
55-
40-
Parkin
70 -
70 -
55 -
PINK1
35 -
15 -
MIF
35 -
20 -
Casp3
Cleaved-Casp3
Ex-time=20s
20 -
Casp3
Cleaved-Casp3
Ex-time=60s
55 -
40 -
35 -
20 -
GAPDH
WB gels of cytoplasmic fraction

Figure 2 Mitochondrial enriched fraction

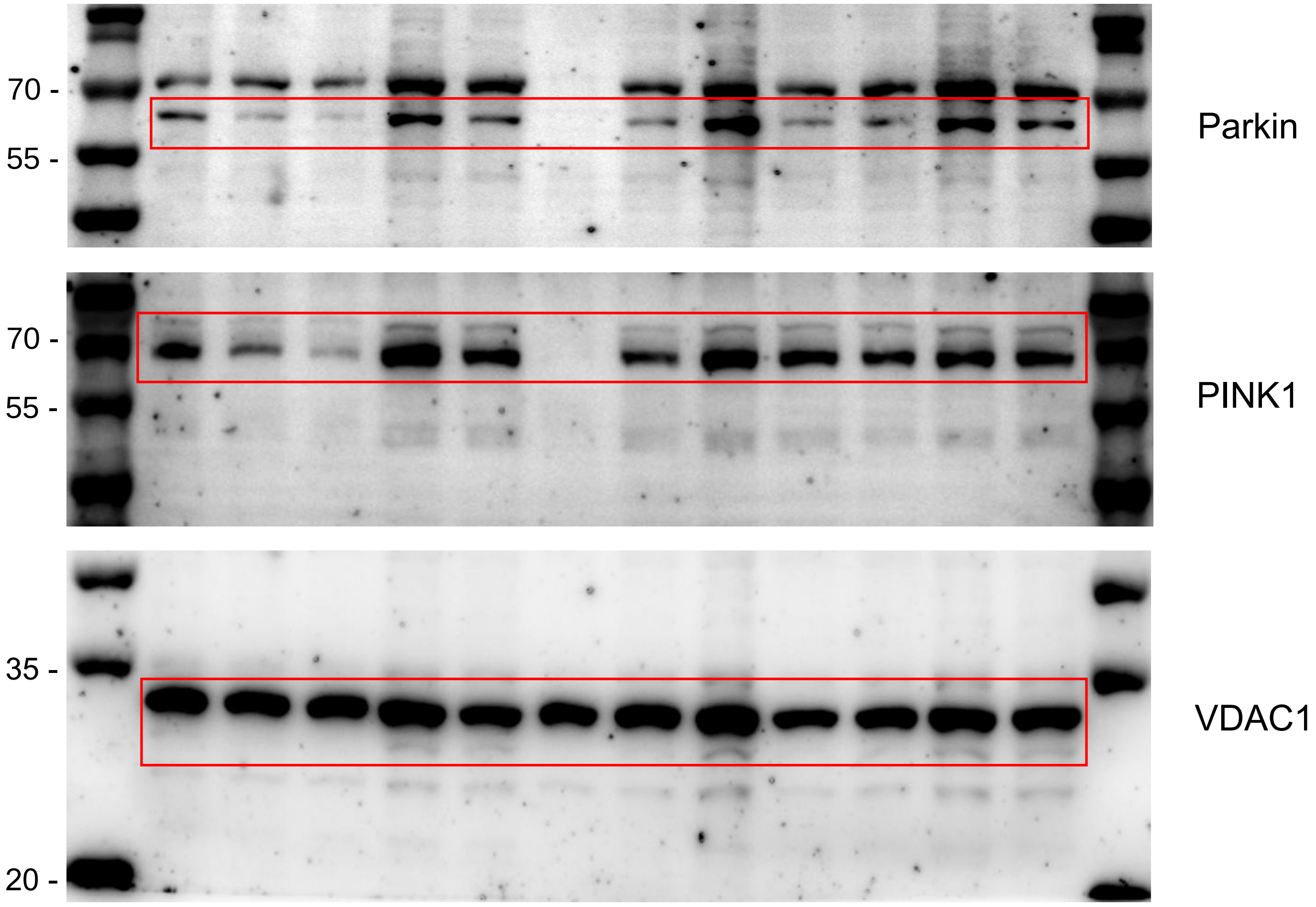

WB gels of mitochondria enriched fraction

Figure 3

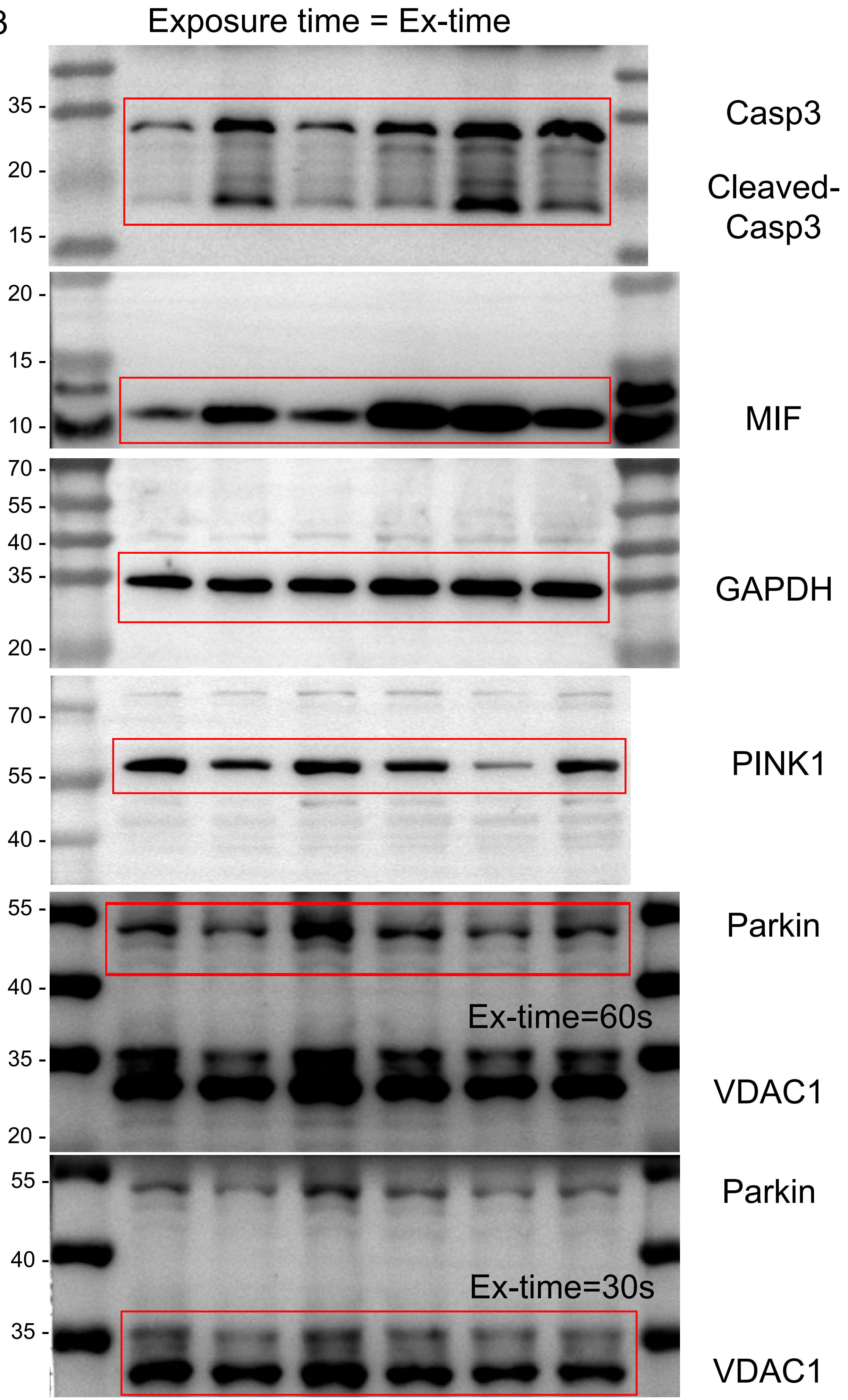

Exposure time = Ex-time
35 -
20 -
15 -
Casp3
Cleaved-Casp3
20 -
15 -
10 -
MIF
70 -
55 -
40 -
35 -
20 -
GAPDH
70 -
55 -
40 -
PINK1
55 -
40 -
35 -
20 -
Parkin
Ex-time=60s
VDAC1
55 -
40 -
35 -
Parkin
Ex-time=30s
VDAC1

Figure 4 co-IP

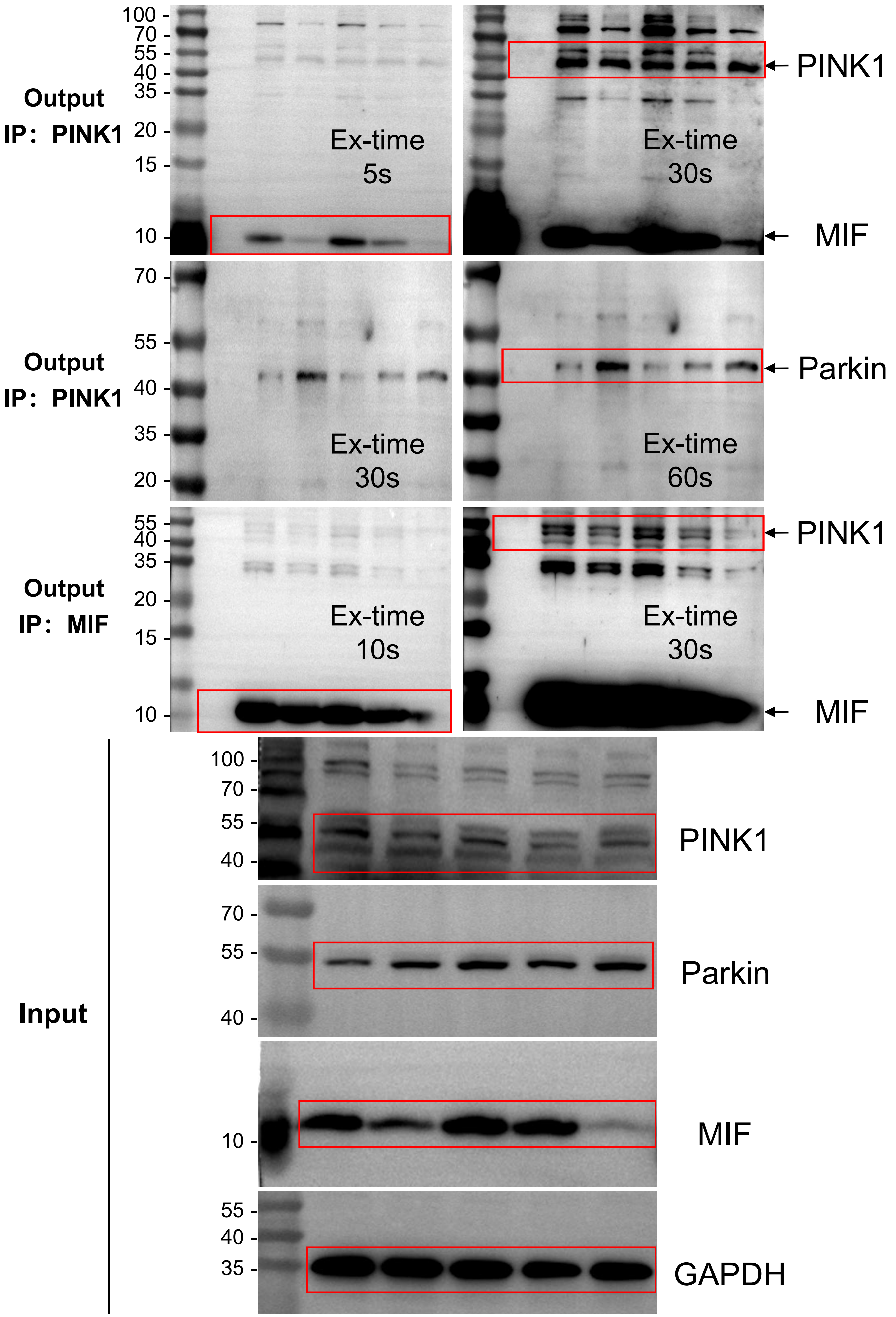

Figure 5

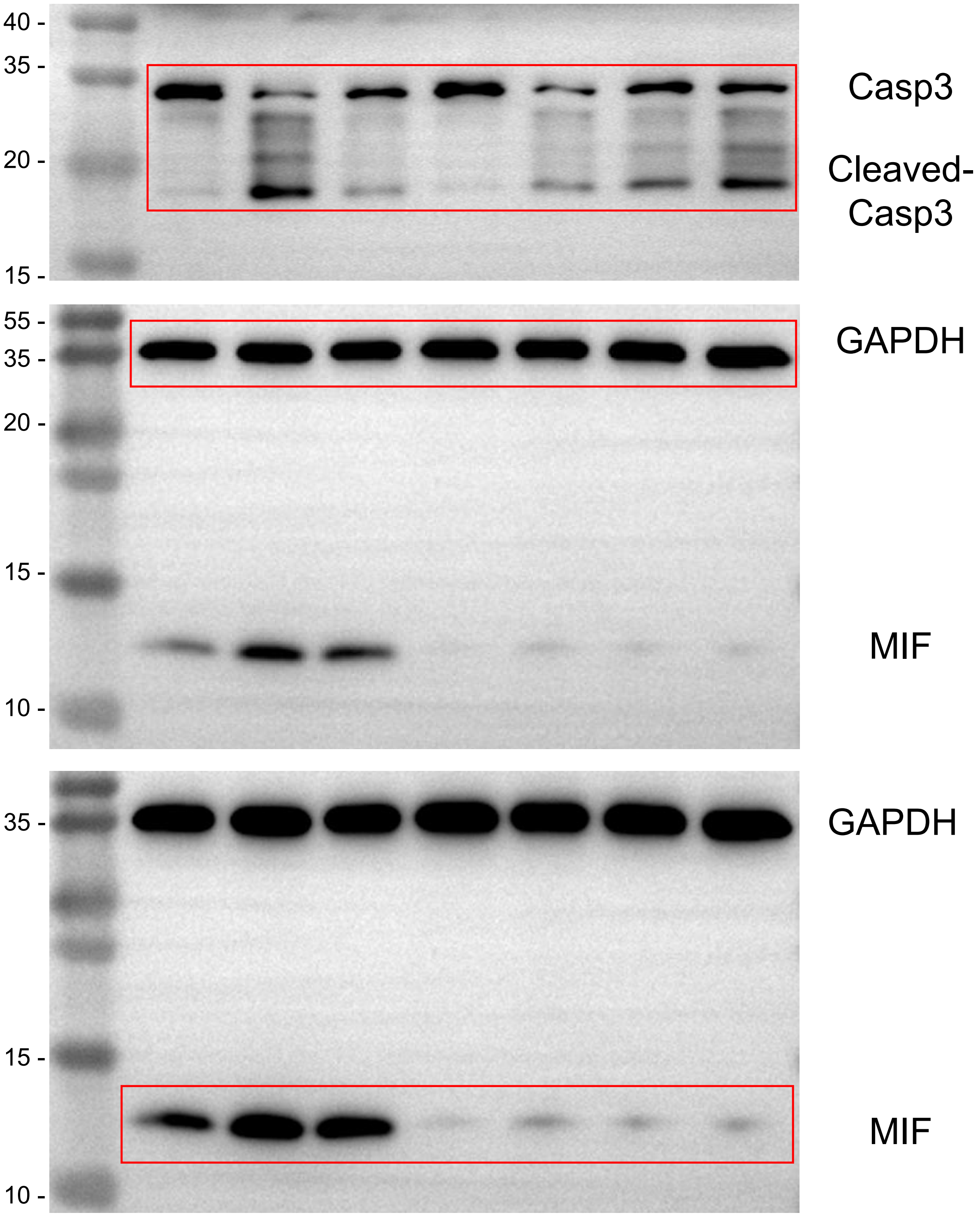
40 -
35 -
20 -
15 -
Casp3
Cleaved-
Casp3
55 -
35 -
20 -
15 -
10 -
GAPDH
MIF
35 -
15 -
10 -
GAPDH
MIF

Figure 5

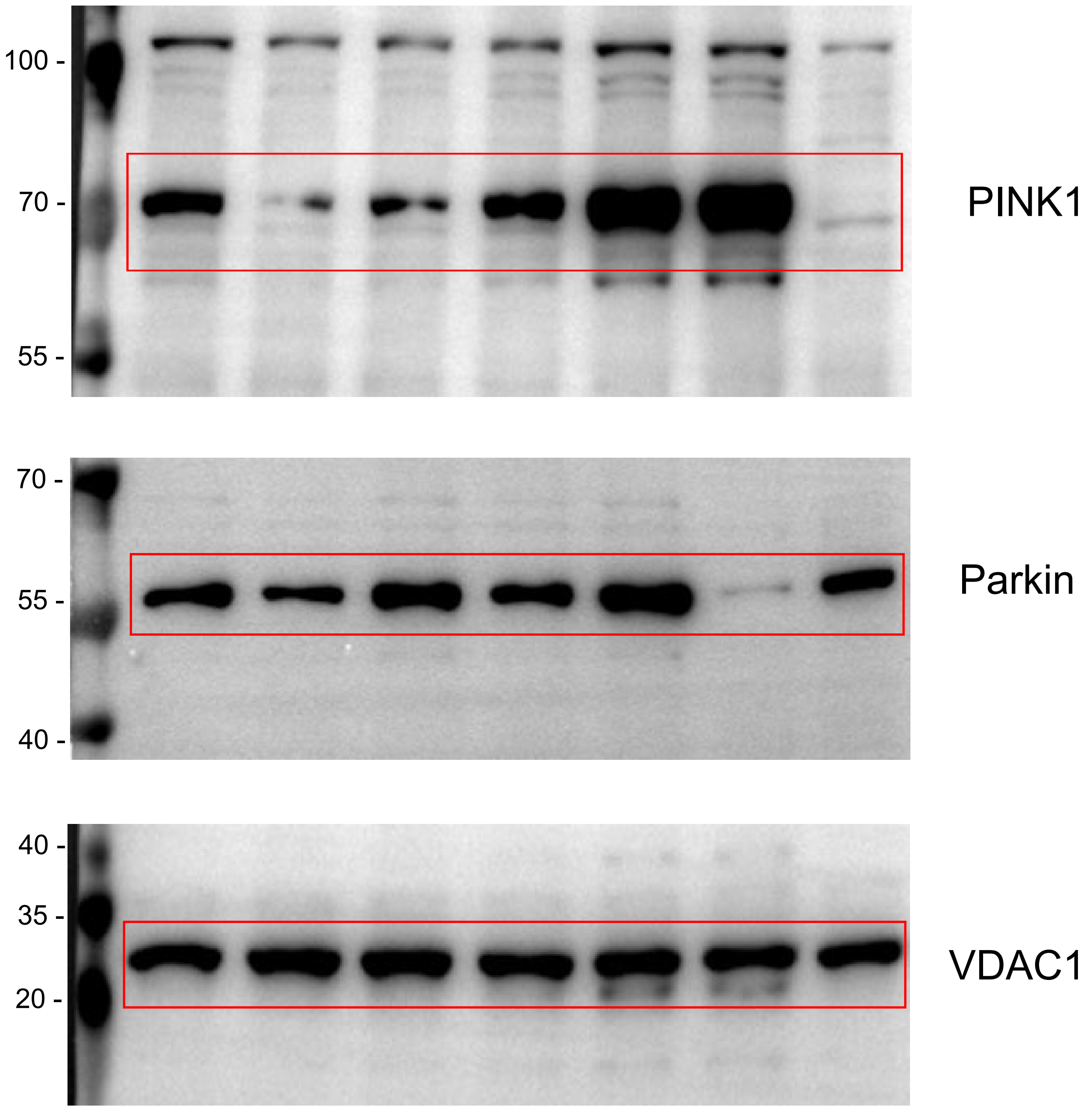

Figure 6

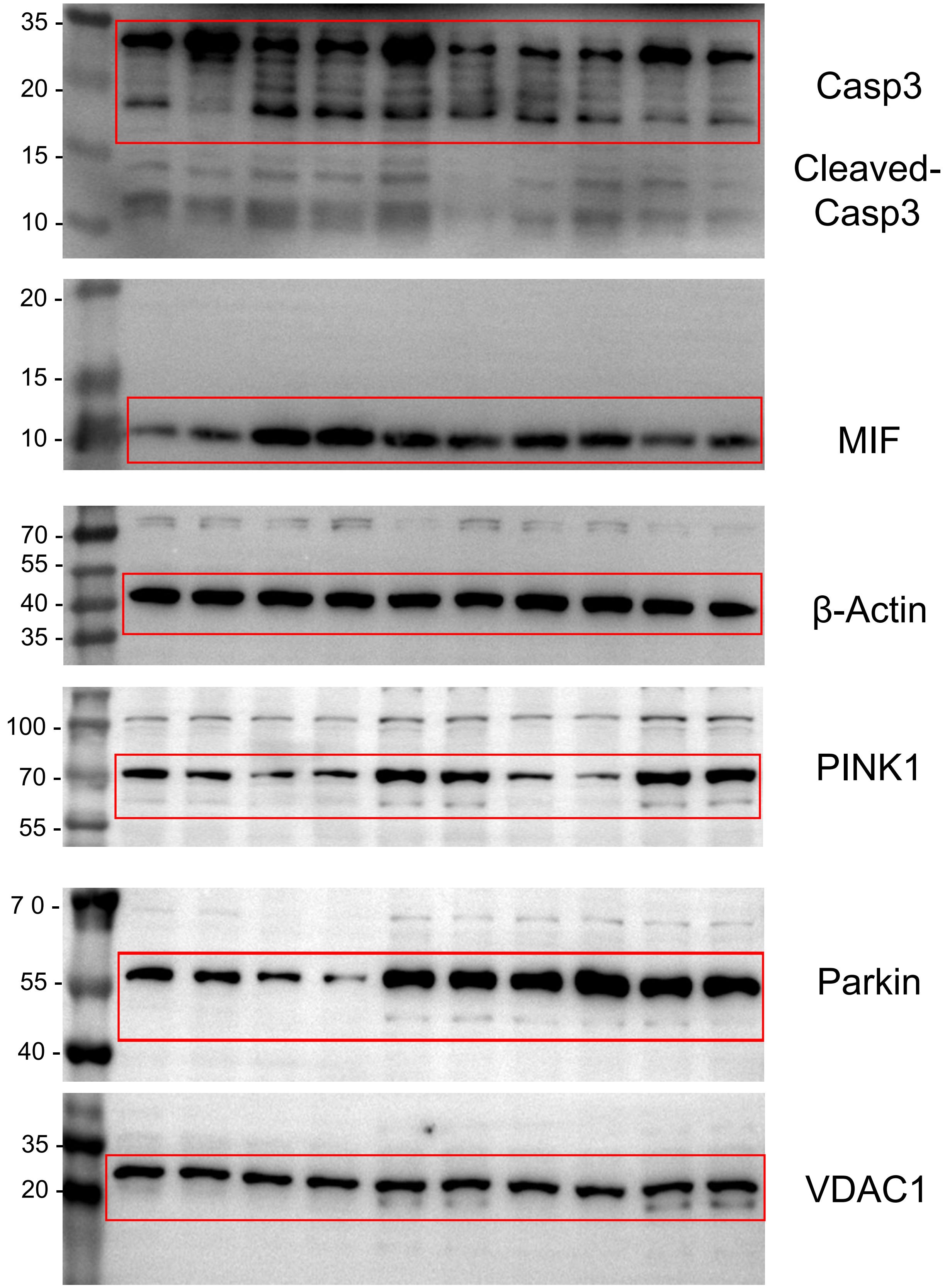

35 -
20 -
15 -
10 -
Casp3
Cleaved-Casp3
20 -
15 -
10 -
MIF
70 -
55 -
40 -
35 -
β-Actin
100 -
70 -
55 -
PINK1
7 0 -
55 -
40 -
Parkin
35 -
20 -
VDAC1

Figure S6

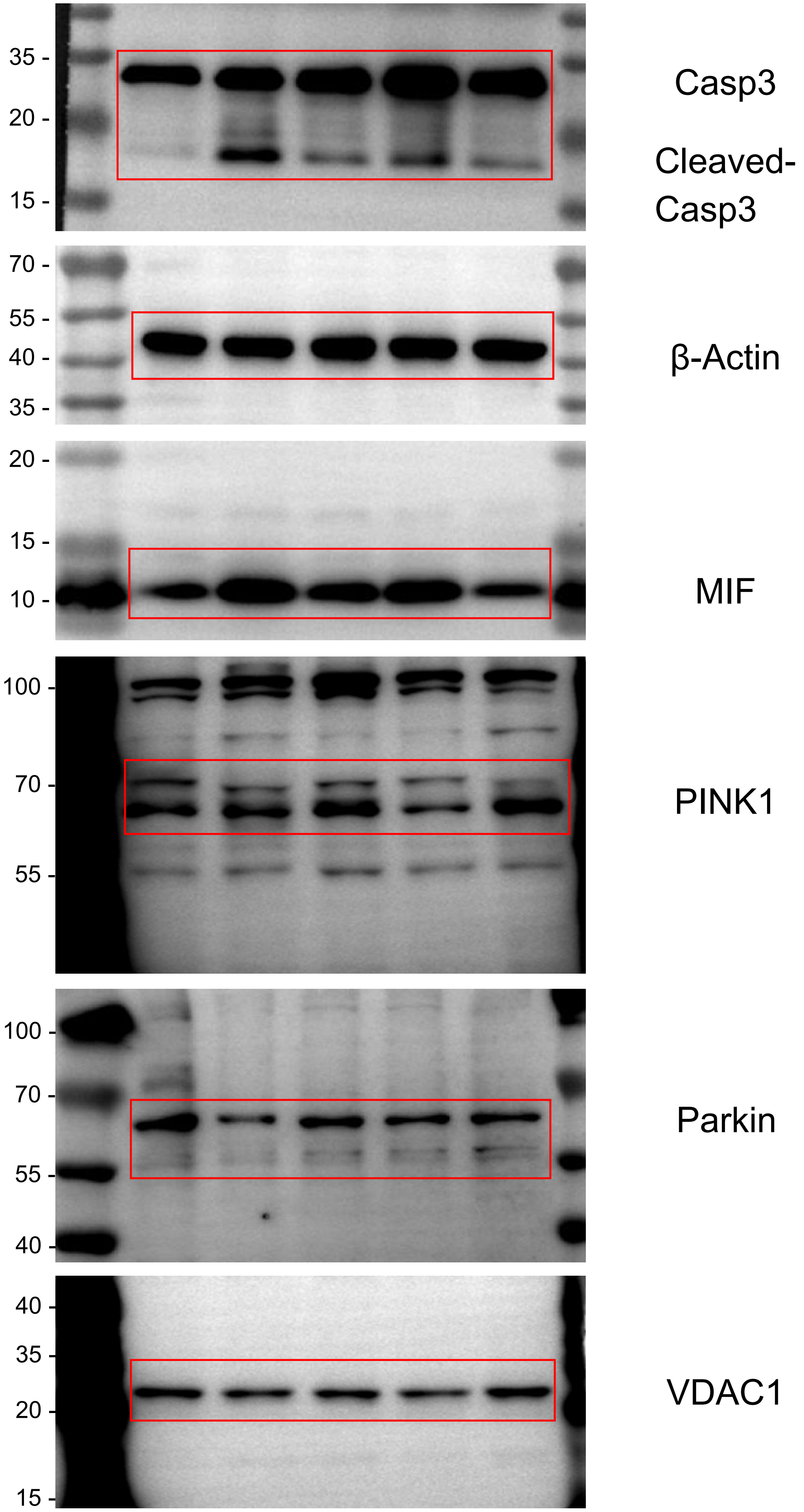
35 -
20 -
15 -
Casp3
Cleaved-Casp3
70 -
55 -
40 -
35 -
β-Actin
20 -
15 -
10 -
MIF
100
70
55
PINK1
100
70
55
40
Parkin
40
35
20
15
VDAC1
