# Supplementary material for: Macrophage migration inhibitory factor (MIF) suppresses mitophagy through disturbing the protein interaction of PINK1-Parkin in sepsis-associated acute kidney injury
Source: Cell Death Dis. 2024 Jul 2;15(7):473. doi: 10.1038/s41419-024-06826-z (PMC11220046; doi:10.1038/s41419-024-06826-z)
Supplement: Supplementary file 2 — Supplementary figure (Figure S6) [file 41419_2024_6826_MOESM2_ESM.pdf]

Supplementary Figure (Figure S6)

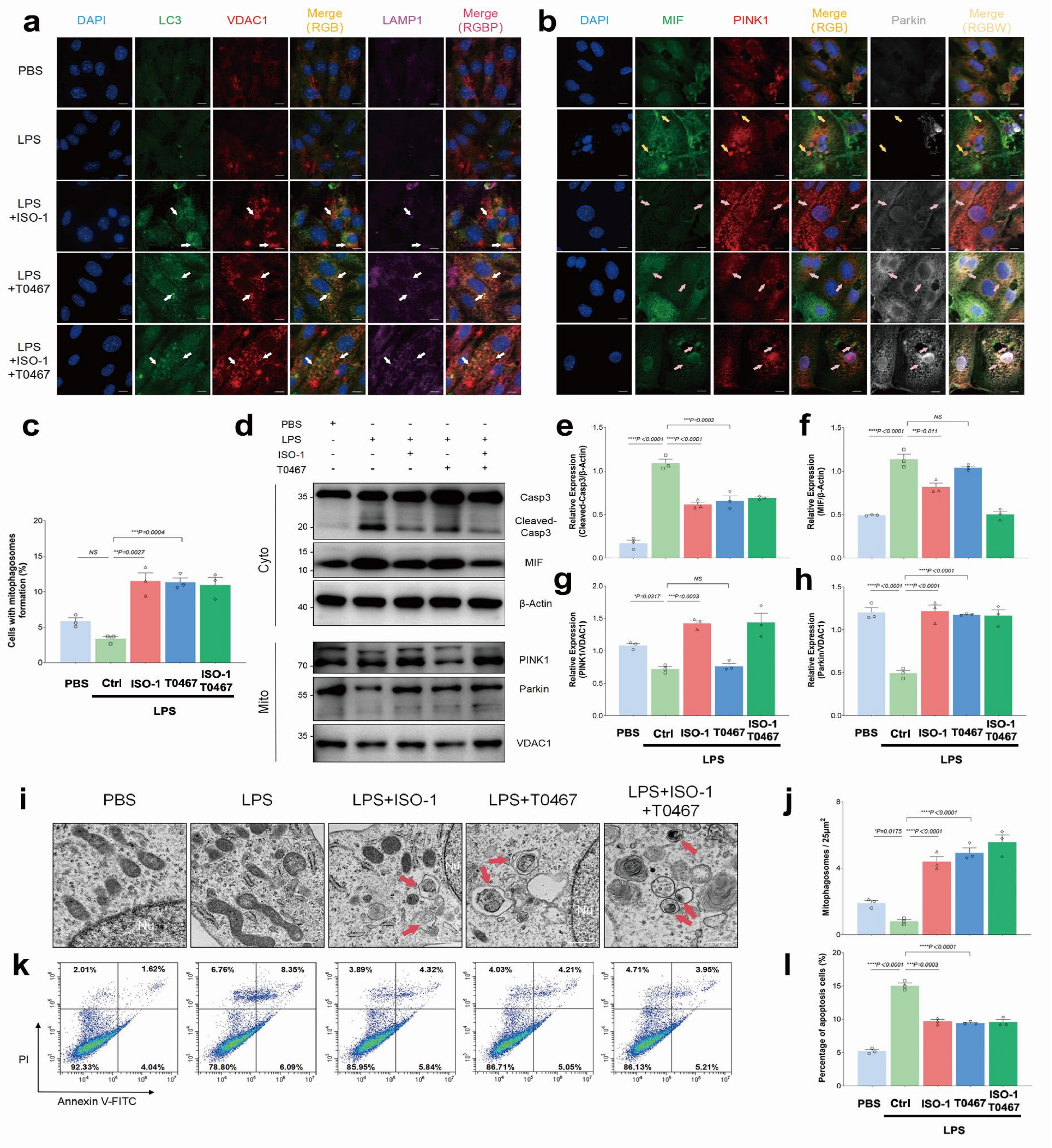

Figure S6: ISO-1 and T0467 exhibited anti-injury properties and activated of PINK1-Parkin-mediated mitophagy in murine primary tubular renal tubular epithelial cells (mPTECs). Represented images of triple-labeling immunofluorescence showed the colocalization of (a) LC3 (green), VDAC1 (red), and Lamp1 (purple), as well as (b) MIF (green), PINK1 (red), adn Parkin (white). The likely mitophagosomes were denoted by white arrows. The colocalization of MIF and PINK1 was marked by a yellow arrow, and the colocalization of PINK1 and Parkin was indicated by a pink arrow. Scale bar: 10 μm. (c) The percantage of mPTECs with mitophagosomes formation was assessed across various groups. (d)The levels of MIF, Casp3, cleaved-Casp3, and β-Actin in the cytoplasmic fraction (Cyto), as well as PINK1, Parkin, and VDAC1 in the mitochondrial enriched fraction (Mito), were determined via immunoblotting. The relative expression ratios of these proteins were quantified: (e) cleaved-Casp3/β-Actin, (f) MIF/β-Actin, (g) PINK1/VDAC1, and (h) Parkin/VDAC1. (i) Representative TEM images depicted the morphology of mitophagosomes (mitochondria-like structures enclosed by autophagosomes, marked by red arrows). Scale bar: 500nm. (j) The mean number of mitophagosomes p er 25 μm^2 was calculated from ten random fields of TEM view. (k,l) Apoptosis in mPTECs, stained with annexin V-FITC and PI, was quantified using FCM. Data were presented as mean ± SEM. n=3. *P＜0.05, **P＜0.01, ***P＜0.001，****P＜0.0001.
